# Contextualization of cost-effectiveness evidence from literature for 382 health interventions for the Ethiopian essential health services package revision

**DOI:** 10.1186/s12962-021-00312-5

**Published:** 2021-09-14

**Authors:** Alemayehu Hailu, Getachew Teshome Eregata, Amanuel Yigezu, Melanie Y. Bertram, Kjell Arne Johansson, Ole F. Norheim

**Affiliations:** 1 Bergen Centre for Ethics and Priority Setting, Department of Global Public Health and Primary Care, University of Bergen, Bergen, Norway; 2 Ministry of Health of Ethiopia, Addis Ababa, Ethiopia; 3 Ethiopian Public Health Institute, Addis Ababa, Ethiopia; 4 Department of Health Systems Governance and Financing, World Health Organization, Geneva, Switzerland; 5 Department of Global Health and Population, Harvard T.H. Chan School of Public Health, Boston, USA

**Keywords:** Cost-effectiveness analysis, Priorities setting, Essential health services package, Ethiopia

## Abstract

**Background:**

Cost-effectiveness of interventions was a criterion decided to guide priority setting in the latest revision of Ethiopia’s essential health services package (EHSP) in 2019. However, conducting an economic evaluation study for a broad set of health interventions simultaneously is challenging in terms of cost, timeliness, input data demanded, and analytic competency. Therefore, this study aimed to synthesize and contextualize cost-effectiveness evidence for the Ethiopian EHSP interventions from the literature.

**Methods:**

The evidence synthesis was conducted in five key steps: search, screen, evaluate, extract, and contextualize. We searched MEDLINE and EMBASE research databases for peer-reviewed published articles to identify average cost-effectiveness ratios (ACERs). Only studies reporting cost per disability-adjusted life year (DALY), quality-adjusted life year (QALY), or life years gained (LYG) were included. All the articles were evaluated using the Drummond checklist for quality, and those with a score of at least 7 out of 10 were included. Information on cost, effectiveness, and ACER was extracted. All the ACERs were converted into 2019 US dollars using appropriate exchange rates and the GDP deflator.

**Results:**

In this study, we synthesized ACERs for 382 interventions from seven major program areas, ranging from US$3 per DALY averted (for the provision of hepatitis B vaccination at birth) to US$242,880 per DALY averted (for late-stage liver cancer treatment). Overall, 56% of the interventions have an ACER of less than US$1000 per DALY, and 80% of the interventions have an ACER of less than US$10,000 per DALY.

**Conclusion:**

We conclude that it is possible to identify relevant economic evaluations using evidence from the literature, even if transferability remains a challenge. The present study identified several cost-effective candidate interventions that could, if scaled up, substantially reduce Ethiopia’s disease burden.

**Supplementary Information:**

The online version contains supplementary material available at 10.1186/s12962-021-00312-5.

## Introduction

Because of the rapid expansion of new technologies and health interventions, priority setting—implicitly or explicitly—is inevitable. To rapidly and efficiently progress towards universal health coverage (UHC), what policy makers can deliberately choose to do is carefully define an optimal national essential health services package (EHSP) that can be delivered within the expected budget envelope [1–5]. Cognizant of this, the Ethiopian government defined its EHSP in 2019, and cost effectiveness was selected as one of the criteria for prioritizing the health interventions in the revision process, together with six other criteria [6].

Ranking interventions by their cost-effectiveness ratio can help prioritize interventions that provide the highest health impact at a relatively lower cost [7]. Many high-income countries and some low- and middle-income countries (LMICs) explicitly use cost-effectiveness analysis (CEA) in policy decisions about the introduction of new interventions into the health system [1, 8, 9]. For example, in Thailand’s’ health technology assessment (HTA) process, CEA is the primary consideration in priority decision of this kind [10]. However, conducting primary health economic evaluations in each of these settings of a wide range of health interventions simultaneously is challenging due to cost, time, scarcity of input data, and computational capacity constraints.

An international effort of donors and academia in support of economic evaluation has produced substantial cost-effectiveness evidence over the past two decades. The World Health Organization (WHO), the Center for the Evaluation of Value and Risk in Health at Tufts Medical Center, and Disease Control Priorities (DCP) have produced cost-effectiveness evidence for priority-setting purposes in LMICs. The Tuft CEA registry is a comprehensive, publicly available database that contains 6,907 cost per quality-adjusted life year (QALY) and 698 cost per disability-adjusted life year (DALY) studies published through 2018 [11]. The DCP-3 synthesized cost-effectiveness ratios for 93 interventions from diverse program areas in 2016 [12]. WHO has produced a series of reports on the cost effectiveness of health interventions targeted in the Millennium Development Goals (i.e., tuberculosis [TB], malaria, HIV/AIDS, and maternal, neonatal, and child health) [13–16]. However, this evidence is mostly at the global or regional level and encompasses limited program areas. Country-specific synthesis and contextualization of cost-effectiveness evidence were therefore necessary for revising the latest Ethiopian EHSP. This paper aimed to synthesize and contextualize the cost-effectiveness evidence for the Ethiopian EHSP interventions from the literature.

## Methods

### Study context

This study was conducted in Ethiopia in 2019 as part of the revision of the Ethiopian EHSP (Box 1) [17]. Ethiopia has a substantial disease burden, with an average life expectancy of 65.5 years [18, 19]. Communicable, maternal, neonatal, and nutritional diseases (CMNNDs) represent the highest disease burden, accounting for 58% of DALY loss in 2017, while noncommunicable diseases (NCDs) accounted for 34% of the disease burden. About 8% of the DALYs were from emergencies and injuries [19]. Furthermore, Ethiopia is a low-income country with a per capita gross domestic product (GDP) of US$953 in 2019 [20]. The per capita health expenditure in Ethiopia in 2016/17 was US$33 [21].

### Identification of relevant health interventions

The detailed steps used to select the interventions are presented elsewhere [6, 22]. From the total of 1018 unique interventions that were considered in the Ethiopian EHSP, the cost-effectiveness ratio was calculated using primarily the WHO-CHOICE GCEA approach for 144 interventions [23]. Additionally, we collected cost-effectiveness evidence for 771 interventions from the literature, excluding 64 multisector nutrition interventions and 39 emergency and critical care interventions [22]. A detailed breakdown of the number of interventions by evidence synthesis method is presented in Table 1.

**Box 1:** Ethiopian EHSP

**What is the EHSP, and why was the revision needed?**
The government of Ethiopia is committed to achieving universal health coverage. Universal health coverage means that every person—no matter who they are, where they live, or how much money they have—should be able to access quality health services without financial hardship.
However, it is impossible to progress toward universal health coverage without clearly identifying the most pressing health problems and what interventions are appropriate to address those health problems efficiently and equitably.
Therefore, defining the essential health services package is the primary step to use the available resources to prioritize the most critical interventions based on cost, equity, financial risk protection, and public interest (community concern) justifications.
An EHSP can be defined as the package of services that the government provides or is aspiring to provide to its citizens equitably.
The Ethiopian EHSP identified the most pressing health challenges and interventions deemed appropriate, affordable, and equitable to address health problems.
**Goal of the EHSP**
To provide access to quality health services for all Ethiopians with full financial risk protection regardless of age, ability to pay, economic status, and residence.
**Objectives of the EHSP**
To reduce the high burden of disease in Ethiopia by making available affordable, high-priority interventions.
To protect the population against catastrophic and impoverishing health expenditures and provide full financial risk protection.
To increase equitable access to health services and interventions.
To increase the efficiency of the health system.
To increase public participation and transparency in decision-making in the health sector.
**The revision process**
The revision process was conducted from May 2018–November 2019.
As recommended by the World Health Organization for designing health benefits packages, the revision was conducted using the best available evidence (data), was based on extensive consultation with all stakeholders (dialogue), and was conducted through an open, transparent, and democratic decision-making process (decision).
Several consultations have been held with public representatives and professional association experts actively participating in the revision process.
Interventions were compared based on seven criteria: disease burden, cost effectiveness, equity, financial risk protection, budget impact, public acceptability, and political acceptability.

**Table 1:** Number of essential health service package interventions and cost-effectiveness evidence synthesis approaches by program area

Major program areas	Total	WHO-CHOICE	Searched	Contextualized
RMNCH	333	51	282	121
Noncommunicable diseases	218	74	144	93
Surgical care	181	0	181	90
Multisectoral nutrition interventions*	64	0	-	-
Major communicable diseases	62	18	44	36
Health education and BCC	57	1	56	13
Emergency and critical care*	39	0	-	-
Neglected tropical diseases (NTDs)	35	0	35	12
Hygiene & environment health (H&EH)	29	0	29	17
Overall	1018	144	771	382

Searched = CEA evidence was sought from the literature; contextualized = CEA evidence was found and contextualized

*BCC*  Behavioral change communication

*For multisectoral nutrition interventions and emergency and critical care interventions, we classified interventions as very cost effective, cost effective, and not cost effective based on local expert judgment

### Evidence synthesis

**Fig. 1:**
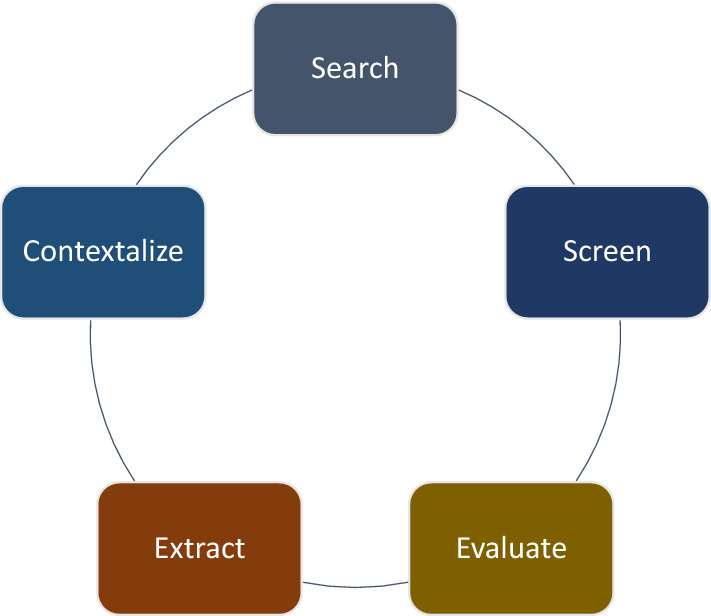
Schematic diagram for the evidence synthesis process ( Source: Produced by the authors for this publication)

We adopted an evidence synthesis strategy developed by the Tuft CEA registry [11]. The cost-effectiveness evidence synthesis was conducted in five key steps: search, screen, evaluate, extract, and contextualize (Fig. 1). The first, second, and third authors (AH, AY, and GTE) conducted all five steps of the evidence synthesis process from January–August 2019.

#### Search

To identify cost-effectiveness studies on a given intervention from the EHSP list, we searched for peer-reviewed and published articles in MEDLINE and EMBASE research databases. These databases are the most used databases for medicine and healthcare evidence synthesis. The search was conducted intervention by intervention using a combination of keywords indicating the intervention name, the program area, and the type of study (i.e., cost effectiveness, cost utility, economic evaluation). For example, for the intervention entitled “Detection of uncomplicated malaria using rapid diagnostic test and treatment with artemether-lumefantrine,” an extensive literature search was conducted using keywords such as “malaria,” “malaria treatment,” “artemether-lumefantrine,” “falciparum,” “vivax,” “rapid diagnosis testing,” “*Plasmodium*,” “cost-effectiveness,”.

#### Screen

In this step, we conducted a preliminary assessment and screening of articles based on the inclusion and exclusion criteria. First, only original studies published in the English language from 1990 through 2019 were included. Second, only economic evaluation studies reporting cost per DALY, QALY, or life years gained (LYG) were included. Priority was given to those studies that reported cost per DALY or QALY, but 28 studies reporting cost per LYG were included. All other articles using a natural unit of measurement (e.g., case identified, cured, or treated) were excluded. Partial economic evaluation studies (e.g., cost of illness) and full economic evaluations using a cost–benefit analysis study were also excluded. Third, we only included studies that compared the intervention with the “doing nothing/null scenario” and studied reporting average cost-effectiveness ratios (ACERs). Fourth, only studies conducted from the health service provider’s perspective were included.

#### Evaluate

The transferability of evidence was thoroughly checked during the evaluation phase by examining the study’s context and its quality. In terms of the study context, studies from low-income settings, particularly from sub-Saharan Africa, were included in the first place. If no study was found in low-income settings, studies from middle- and high-income settings were also included as an alternative.

The final appraisal of the transferability and quality of studies was done using the Drummond checklist [24]. The Drummond checklist has 10 domains, and we scored each domain as 0 or 1 (0 = not fulfilled and 1 = fulfilled) with an aggregate score out of 10 points. Only studies with a score of at least 7 were included (Additional file 1). When multiple studies were found on the same interventions, recent studies and those with a higher quality score were included. For the purpose of quality control, all the articles were double checked by two reviewers. All the studies were exported to EndNote reference managing software to avoid duplication. The full list of studies with the score is provided in Additional file 2.

#### Extract

Once the high-quality cost-effectiveness studies were identified, the extraction of the information from the articles was done using a predefined data extraction format (Additional file 3). The data extraction format contains the country of the study, base year, currency, type of ratio reported (i.e., ICER, ACER, or both), unit cost, total cost, and discounts. We extracted the following information from each of the studies: ACER, country, base year of analysis, currency, units of health outcome measurement (i.e., DALY, QALY, or LYG), unit cost per intervention, total cost, and total DALY/QALY/LYG. We also extracted information about whether or not discounting was done and, if done, what percentage of discounting for cost and health outcome was applied.

#### Contextualize

Contextualization of the information was done by adjusting the currency and time differences across the individual studies. First, an appropriate exchange rate was used to convert the currencies from local currency units into US$ [25]. Then, to convert the ACERs reported in various years into 2019 US$, we employed the US GDP deflator. Finally, all the ACERS are reported in 2019 US$. Studies from a country where context varied too much from the Ethiopian setting were excluded at this stage.

### Data analysis

Descriptive analysis was done to summarize the findings for each of the interventions into program areas. We initially generated the median ACER with interquartile range (IQR) across the program. The results are presented in tabular, bar graph, and dot-plot forms. We also present the ACERs in the form of a league table. The data were analyzed using Stata version 16 and Microsoft Excel.

## Results

In total, ACERs for 382 interventions were synthesized from seven major program areas. The ACERs were collected from 268 studies. Many of the included studies were conducted in the period 2010–2014 (38%), with fewer in the period 2015–2018. The majority (57%) of the studies were from LMICs in Africa or other LMICs outside of Africa (e.g., Pakistan, China, Thailand). We found an ACER for 13 interventions from study sources in Ethiopia. In comparison, 43% were from high-income countries. Most (32%) of the interventions are from the reproductive maternal neonatal and child health (RMNCH), followed by NCDs (24%), surgical care (23%), communicable diseases (CD) (9%), and hygiene and environmental health interventions (5%).

The majority (68%) of the studies included were scored 10 out of 10 based on the Drummond checklist. Nearly half (46%) of the studies employed DALY as a health outcome measure while the other 45% employed QALY and 7% employed LYG. We present the full list of ACERs for interventions by program area and sub-program area in the Additional file 3. In Table 2 below, we present the key findings for major program areas.

**Table 2:** Summary of contextualized studies

Characteristics	Number	Percentage (%)
Study periods (n = 382)		
1990–1994	10	3%
1995–1999	15	4%
2000–2004	71	19%
2005–2009	98	26%
2010–2014	144	38%
2015–2018	44	12%
Study regions (n = 382)		
LMIC in Africa	173	45%
LMIC outside Africa	44	12%
United States of America	73	19%
United Kingdom	32	8%
Other high-income countries	60	16%
Health outcome measures (n = 382)		
DALY	174	46%
QALY	180	47%
LYG	28	7%
Major program area (n = 382)		
RMNCH	121	32%
Surgical care	90	23%
NCD	93	24%
CD	36	9%
H&EH	21	5%
NTD	12	3%
HE & BCC	13	3%
Quality score of the studies (n = 268)		
Score 10/10	183	68%
Score 9/10	56	21%
Score 8/10	26	10%
Score 7/10	3	1%

**Fig. 2:**
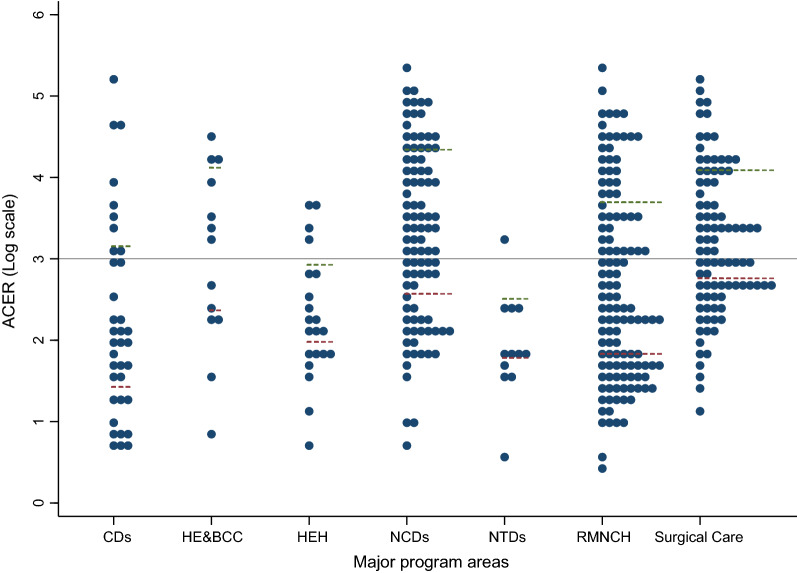
The ACERs for 382 health interventions by major program area. The Y-axis is ACER in the log scale. The horizontal gray line represents ACER = US$1000 per DALY/QALY/LYG. A dot represents an ACER for a single intervention

An overview of the ACERs for interventions by major program area is presented in Fig. 2. The Y-axis represents ACER in the log scale. A dot represents an ACER for a single intervention. The horizontal gray line represents ACER = US$1,000 per DALY. Overall, slightly more than half of the interventions had ACERs of less than US$1,000 (n = 216; 56%). However, the majority of NTDs (n = 11; 92%), hygiene and environmental health (n = 17; 81%), and communicable disease (n = 27; 75%) had ACERs lower than US$1,000 while less than half (n = 37; 40%) of NCD interventions had ACERs below US$1,000.

**Fig. 3:**
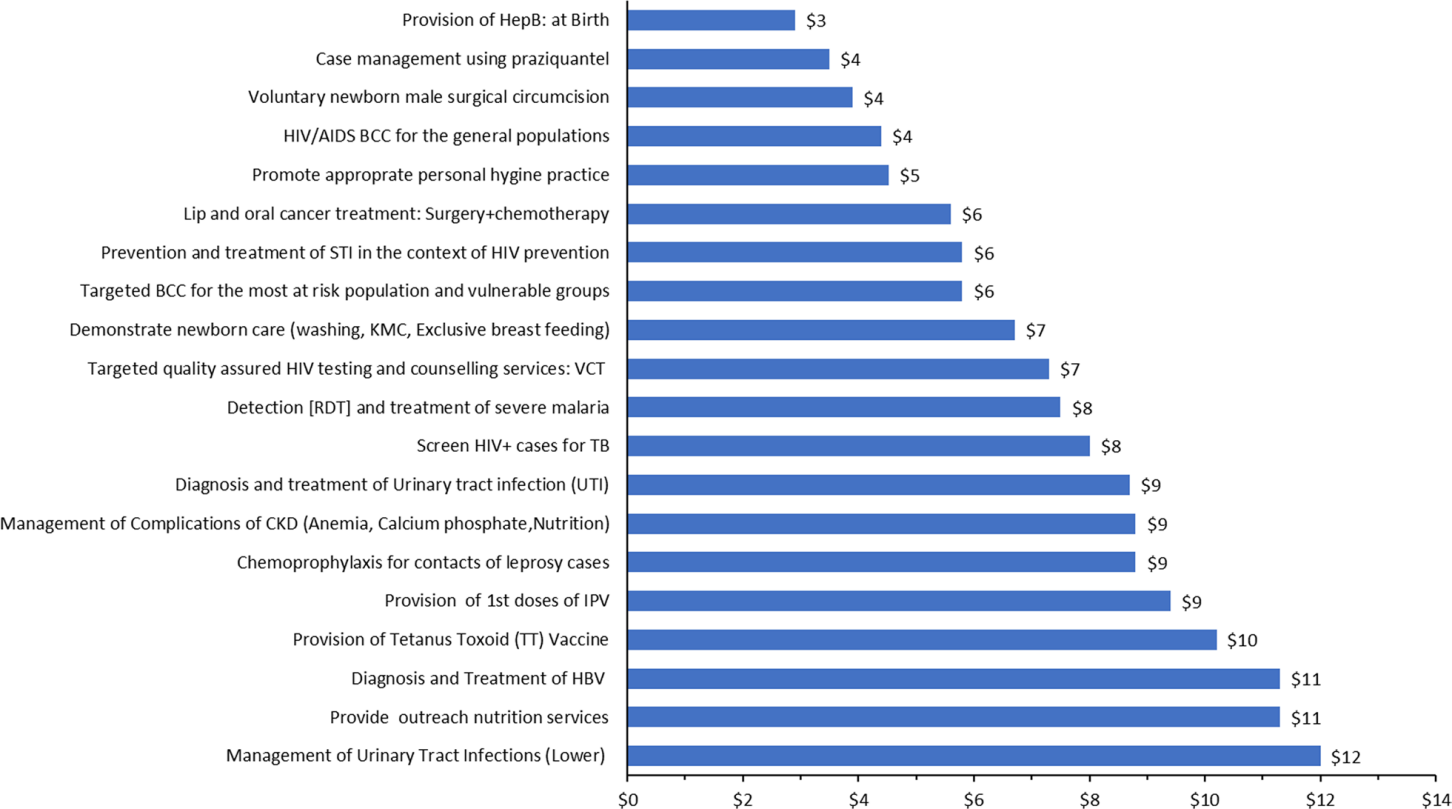
The Average cost-effectiveness ratio (ACER) for the 20 most cost-effective interventions (in US$ per DALY/QALY/LYG)

**Fig. 4:**
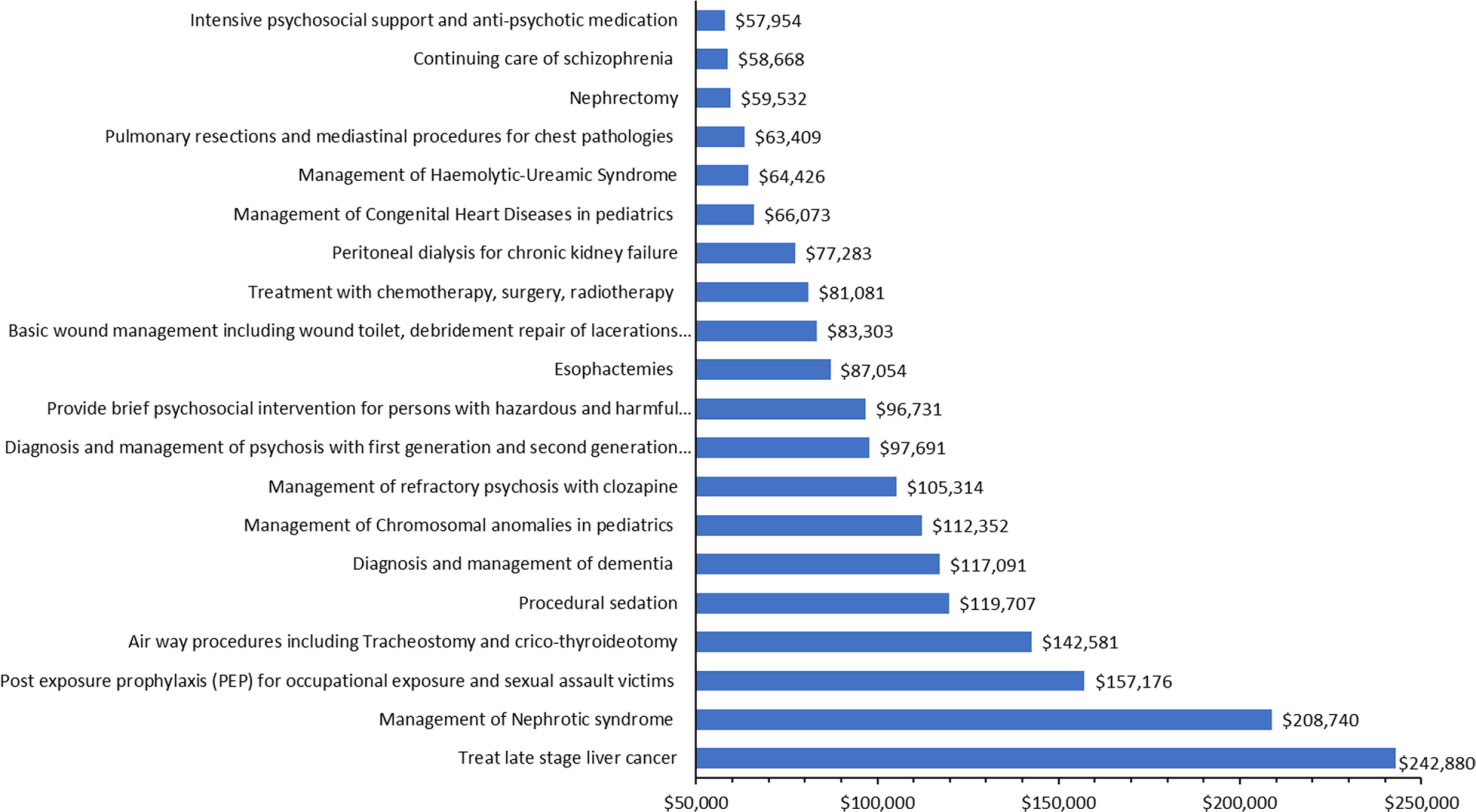
The Average cost-effectiveness ratio (ACER) for the 20 least cost-effective interventions (in US$ per DALY/QALY/LYG)

In general, we found ACERs ranging from the lowest of US$3 per DALY averted (for the provision of hepatitis B vaccination at birth) to the highest of US$242,880 per DALY averted (for late-stage liver cancer treatment). Figure 3 presents an overview of the 20 most cost-effective interventions, and Fig. 4 shows the 20 least cost-effective interventions based on the ACER ranking. In both the top 20 and bottom 20 interventions, we found that many of the major program areas were represented. We present the full list of ACERs for interventions by program area and sub-program area in the Additional file 3. In Table 3, we present the range, median, and IQR of ACERs by major program area. The overall median of the ACERs was 677 (IQR: 87–4761).

**Table 3:** Descriptive summary of the average cost- effectiveness ratios (in US$ per DALY/QALY/LYG) for health interventions by program (n = 382)

Program area	Median	p25	p75	Min	Max	N	Major program
Maternal health	24	19	131	13	21,757	8	RMNCH
NTDs	65	44	231	4	1675	12	NTDs
Leprosy	74	32	116	9	138	4	CDs
Malaria	81	35	609	8	1185	8	CDs
HIV/AIDS	116	7	854	4	157,176	15	CDs
H&EH	116	59	183	5	1665	17	H&EH
Eye health problems	273	134	687	78	949	6	NCDs
SRH	273	166	2812	9	52,747	26	RMNCH
Nutrition	311	52	1158	11	56,792	35	RMNCH
Child health	395	32	18,696	3	208,740	40	RMNCH
Newborn health	510	92	3751	4	16,460	12	RMNCH
Diabetes mellitus	1005	827	6356	60	9450	6	NCDs
Surgical care	1101	400	5506	14	142,581	82	Surgical care
CVD	1198	122	4463	46	48,729	26	NCDs
STI	1298	20	9302	18	49,968	9	CDs
Cancer	1520	140	20,523	6	242,880	27	NCDs
Health education	1742	167	9413	7	33,763	13	HEBCC
CRD	7564	4112	20,420	164	20,533	5	NCDs
Anesthesia	9287	1598	17,688	184	119,707	8	Surgical care
Renal diseases	13,930	2120	55,625	9	77,283	7	NCDs
MNSUD	20,606	1257	77,699	209	117,091	16	NCDs
Overall	677	87	4761	3	242,880	382	Overall

*CVD *cardiovascular diseases, *CRD* chronic respiratory diseases, *H&EH* hygiene & environmental health, *MNSUD* mental, neurological, & substance use disorders, *SRH* sexual and reproductive health, *STI* sexually transmitted infections, *N* number of interventions, *p25* first quintile, *p75* third quintile, *min* minimum, *max* maximum

## Discussion

We contextualized cost-effectiveness evidence for a relatively comprehensive list of interventions to the Ethiopian context for the revision of the country’s EHSP. In this study, we found that, while most CDs, NTDs, and hygiene and environmental health interventions had relatively low ACERs, more than half of the NCD interventions had an ACER higher than US$1,000 per DALY. Compared with the need for the purpose of EHSP revision, the amount of cost-effectiveness evidence available in the literature so far is limited in all program areas. It is critically scarce in some programs, such as multisectoral interventions, emergency and critical care, and surgical care. These findings on the extent of the available evidence and the variation in ACERs across program areas or disease categories are similar in many ways to the findings of Tufts’ Global Health Cost-Effectiveness Analysis Registry [26].

The availability of cost-effectiveness evidence customized to the epidemiological and socioeconomic context of the country is a central element in the proper revision of the EHSP. However, our findings show that only a few cost-effectiveness studies exist for a specific country in Africa. For example, we found an ACER for only 13 interventions in studies from Ethiopia, eight from Kenya, seven from Malawi, six from Tanzania, five from Uganda, and four from Zambia. A recent analysis of Tufts Medical Center’s CEA registry indicates the same [27]. Furthermore, as was agreed upon in preparing the roadmap for revising the Ethiopian EHSP, we included studies conducted from a health systems perspective and studies reporting ACERs [6]. This further limited the number of studies available per country. Therefore, to generate more transferable cost-effectiveness evidence across countries, primary cost-effectiveness studies should be expanded in all of Africa. This challenge can be addressed partly by training health economists and public health practitioners on the economic evaluation of health interventions in Africa [28].

In the screening step (Fig. 1), we use ‘null scenario’ as a comparator. The null scenario is a counterfactual scenario that assumes none of the interventions existed (i.e., zero cost and zero benefits). Therefore, the use of ‘null scenario’ as a comparator allows policymakers to broadly compare the ACERs across wide ranges of program areas—within the health sector (i.e., a sector-wide analysis) [29]. Studies that employed “status quo/current practice” as comparators were excluded. Using the status quo or current practice as a comparator implicitly assumes that the current resource use is efficient, while this may not be the case. Comparison of incremental cost-effectiveness ratios (ICERs) using the “current practice” approach is therefore restricted within a group of specific health interventions [8, 29–32].

This study has some limitations, and the findings should be interpreted carefully. First, some relevant cost-effectiveness studies might be excluded because of the relatively stringent screening criteria employed in this study. Based on the protocol agreed upon by all the stakeholders for the revision of the Ethiopian EHSP, we included only economic evaluation studies with cost-per-DALY, -QALY or -LYG measures [6]. Thus, economic evaluations with the cost–benefit ratio as well as cost per natural unit studies were excluded. However, a bibliometric analysis of published economic evaluation studies by Pitt et. al suggests that cost-utility analyses account for at least half of economic evaluations [33], and other costs per natural unit of measurement may be informative in terms of guiding decisions within a specific program.

Second, the variability in terms of the quality of the studies and transparency in the reporting of cost and health impacts was another challenge to this analysis [26]. Although we employed the Drummond checklist to evaluate the quality of the studies uniformly, there could be some high-quality studies excluded or vice versa. There is some variability in the detailed costing and health benefits measurement approaches. For example, while some of the studies employed a top-down costing, some of the studies were based on ingredients costing. Similarly, while some of the studies used a randomized trial setting to measure intervention benefits, some of them were based on pragmatic clinical or population-based cross-sectional studies. Furthermore, many of the included studies were from countries and health system contexts substantially different from Ethiopia. Therefore, we recommend that a further detailed examination of individual studies would improve the transferability of the studies [34, 35].

Third, this study is not a full systematic review. The ACERs were obtained from the best available individual studies. Further analytic work (e.g., meta-analysis and pooled systematic reviews) on a specific intervention or a group of intervention is needed [36, 37]. Furthermore, we recommend that a formal HTA body should be institutionalized in Ethiopia that can conduct a full-scale assessment of intervention costs and benefits. Cost-effectiveness databases should be established in Ethiopia to regularly examine the evidence gap and feed strategic information to the Ministry of Health, Health Insurance Agency, and Ethiopian Pharmaceutical Supply Agency in a timely way.

Fourth, nearly half (48%) of the studies used in this analysis are from high-income countries settings. Since the context in which the intervention’s cost and effectiveness are evaluated varies from the Ethiopian settings, the ACERs also vary. For example, the human resource cost in Ethiopia is relatively low compared with high-income countries in general [38]. Hence, careful consideration should be taken when interpreting the ranking in the league table, and ACERs should be taken only as a general guide in the priority setting process. In addition, a methodological tool is needed that can facilitate the transferability of cost-effectiveness evidence across jurisdictions. There is limited methodological guidance on how to conduct transferability of cost-effectiveness studies across settings [39, 40]. Most importantly, more cost-effectiveness studies should be conducted in Ethiopia, and other low-income settings, using country-level data.

The fifth limitation is that only articles published in the English language were included; we had limited information about cost-effectiveness ratios from articles published in other languages. Additionally, unpublished program evaluation reports were not included in this study, and therefore there may be a publication bias in our data. It is likely that the unpublished reports tend to have more negative findings (i.e., “not cost effective’) than published articles [10].

## Conclusion

In conclusion, it is possible to identify relevant economic evaluations using evidence from the literature, even if transferability remains a challenge. The present study identified several cost-effective candidate interventions that could, if scaled up, substantially reduce Ethiopia’s disease burden. However, there are gaps in the available evidence on cost effectiveness that can be closed only by conducting more economic evaluation research in developing countries. Therefore, we recommend a concerted effort to establish country-level and a multi-country cost and cost-effectiveness databases in Africa. Furthermore, capacity building through the training of health economists in Africa should be widely undertaken.

## Supplementary Information

**Additional file 1.** Example of how evaluation of the studies was done.

**Additional file 2.** Evaluation of studies.

**Additional file 3.** ACER for 382 EHSP interventions.

## Data Availability

The data sets supporting the conclusions of this article is fully available in the manuscript and additional files.

## Abbreviations

ACER: Average cost-effectiveness ratios
BCC: Behavioral change communication
CD: Communicable diseases
CEA: Cost-Effectiveness analysis
CMNNDs: Communicable, maternal, neonatal, and nutritional diseases
CRD: Chronic respiratory diseases
CVD: Cardiovascular diseases
DALY: Disability-adjusted life year
DCP: Disease control priorities
EHSP: Essential health service package
GDP: Gross domestic product
H&EH: Hygiene & environmental health
HTA: Health technology assessment
ICER: Incremental cost-effectiveness ratio
IQR: Interquartile range
LMICs: Low- and middle-income countries
LYG: Life years gained
MNSUD: Mental, neurological, & substance use disorders
NCD: Noncommunicable diseases
NTDs: Neglected tropical diseases
QALY: Quality-adjusted life year
RMNCH: Reproductive maternal neonatal and child health
SRH: Sexual and reproductive health
STI: Sexually transmitted infections
UHC: Universal health coverage
US$: United States Dollar
WHO: World Health Organization
