# Supplementary material for: Contextualization of cost-effectiveness evidence from literature for 382 health interventions for the Ethiopian essential health services package revision
Source: Cost Eff Resour Alloc. 2021 Sep 14;19:58. doi: 10.1186/s12962-021-00312-5 (PMC8442298; doi:10.1186/s12962-021-00312-5)
Supplement: Supplementary file 1 — Additional file 1. Example of how evaluation of the studies was done. [file 12962_2021_312_MOESM1_ESM.pdf]

Supplement 1: Example of how evaluation of the studies using the Drummond checklist was done

160. A Cost-Effectiveness Analysis of a Program to Control Rheumatic Fever and Rheumatic Heart Disease in Pinar del Rio, Cuba (https://doi.org/10.1371/journal.pone.0121363)			
S. No	Checklist	Score	Description
1	Was a well-defined question posed in answerable form?	Yes (1)	A well-defined research question was posed. The objective of the study was to evaluate the cost-effectiveness of a comprehensive effort to control ARF/RHD (primary prevention, secondary preventions, treatment using surgical intervention for RHD)
2	Was a comprehensive description of the competing alternatives given?	Yes (1)	Competing alternatives were described clearly
3	Was the effectiveness of the programme established?	Yes (1)	The effectiveness of the program was established using evidence from previous studies
4	Were all the important and relevant costs and consequences for each alternative identified?	Yes (1)	Important and relevant cost and consequence identified
5	Were costs and consequences measured accurately in appropriate physical units?	Yes (1)	Both costs and health outcomes were valued appropriately. Cost measure includes program cost (major components: administration, education, surveillance, and external evaluation) and direct medical cost. Health outcome was measured using DALY
6	Were costs and consequences valued credibly?	Yes (1)	Costs and health benefits were valued credibly. A standard Global Burden of Disease approached was employed for the measurement of health outcome. The cost was measured from a provider perspective.
7	Were costs and consequences adjusted for differential timing?	No (0)	Lifetime horizon was used. But both cost and health outcomes were not discounted
8	Was an incremental analysis of costs and consequences of alternatives performed? Was an average cost per DALY or QALY provided (comparison with 'doing-nothing') *	Yes (1)	the 10-year Cuban program on RHD was evaluated compared with a "do nothing" approach
9	Was allowance made for uncertainty in the estimates of costs and consequences?	Yes (1)	Uncertainty analysis using Monte Carlo simulations and several scenario analyses exploring the impact of alternative assumptions about the program's effects and costs was done.
10	Did the presentation and discussion of study results include all issues of concern to users?	Yes (1)	The results are presented and discussed in detail, including all major issues.
	Overall score	9	Accepted

*Modified from the original Drummond checklist that the studies reporting average cost-effectiveness ratio or studies comparing the "intervention" against 'doing-nothing' were included.
