# Supplementary material for: Contextualization of cost-effectiveness evidence from literature for 382 health interventions for the Ethiopian essential health services package revision
Source: Cost Eff Resour Alloc. 2021 Sep 14;19:58. doi: 10.1186/s12962-021-00312-5 (PMC8442298; doi:10.1186/s12962-021-00312-5)
Supplement: Supplementary file 2 — Additional file 2. Evaluation of studies. [file 12962_2021_312_MOESM2_ESM.pdf]

Supplement file 2: Evaluation of studies used for the synthesis of cost-effectiveness evidence for the Ethiopian essential health service package using Drummond 10 point cheaklist.

C1= Well-defined question? C2 = Description of alternatives? C3 = Effectiveness establishment? C4 = All relevant costs and consequences? C5 = Appropriate measurement? C6 = Credible valuation? C7 = Differential timing? C8 = Incremental costs and consequences? C9 = Allowance made for uncertainty? C10 = All results concerned to users?

Ref.	Authors, Year	C1	C2	C3	C4	C5	C6	C7	C8	C9	C10	SC
[1]	(Jha, Bangoura et al. 1998)	1	1	1	1	1	1	1	0	0	1	8
[2]	(Stover, Rosen et al. 2017)	1	1	1	1	1	1	1	1	1	1	10
[3]	(Mvundura, Nundy et al. 2015)	1	1	1	1	1	1	1	0	0	1	8
[4]	(Sonnenberg, Burkman et al. 2004)	1	1	1	1	1	1	1	1	1	1	10
[5]	(Fragoulakis, Kourlaba et al. 2012)	1	1	1	1	1	1	1	1	1	1	10
[6]	(Hu, Grossman et al. 2009)	1	1	1	1	1	1	1	1	1	1	10
[7]	(Garside, Stein et al. 2004)	1	1	1	1	1	1	1	1	1	1	10
[8]	(Campos, Sharma et al. 2017)	1	1	1	1	1	1	1	1	1	1	10
[9]	(Aledort, Hook et al. 2005)	1	1	1	1	1	1	0	1	1	1	9
[10]	(Looker, Wallace et al. 2015)	1	1	1	1	1	1	1	1	1	1	10
[11]	(Hansen and Chapman 2008)	1	1	1	1	1	1	1	1	1	1	10
[12]	(Eells, Bharadwa et al. 2013)	1	1	1	1	1	1	0	1	1	1	9
[13]	(Cooper, Shepherd et al. 2012)	1	1	1	1	1	1	1	1	1	1	10
[14]	(Hogan, Baltussen et al. 2005)	1	1	1	1	1	1	0	1	1	1	9
[15]	(Chola, McGee et al. 2015)	1	1	1	1	1	1	0	1	1	1	9
[16]	(Burgos, Gaebler et al. 2010)	1	1	1	1	1	1	1	1	1	1	10
[17]	(Norman, Spencer et al. 2010)	1	1	1	1	1	1	1	1	1	1	10
[18]	(Juillard, Smith et al. 2015)	1	1	1	1	1	1	0	1	1	1	9
[19]	(Willcox, Moorthy et al. 2019)	1	1	1	1	1	1	1	1	1	1	10
[20]	(Adam, Lim et al. 2005)	1	1	1	1	1	1	0	1	1	1	9
[21]	(Shmueli, Meiri et al. 2012)	1	1	1	1	1	1	1	1	1	1	10
[22]	(Lohse, Marseille et al. 2011)	1	1	1	1	1	1	1	0	1	1	9
[23]	(Profit, Lee et al. 2010)	1	1	1	1	1	1	1	1	1	1	10
[24]	(Corlew, Alkire et al. 2016)	1	1	1	1	1	1	1	1	1	1	10
[25]	(Cardarelli, Vaikunth et al. 2018)	1	1	1	1	1	1	1	1	0	1	9
[26]	(Ochalek, Revill et al. 2018)	1	1	1	1	1	1	0	1	0	1	8
[27]	(Antillon, Bilcke et al. 2017)	1	1	1	1	1	1	1	1	1	1	10
[28]	(Xie, da Silva et al. 2012)	1	1	1	1	1	1	0	1	1	1	9
[29]	(Baltussen and Smith 2012)	1	1	1	1	1	1	1	1	1	1	10
[30]	(Vossius, Lotto et al. 2014)	1	1	1	1	1	1	1	1	1	1	10

Ref.	Authors, Year	C1	C2	C3	C4	C5	C6	C7	C8	C9	C10	SC
[31]	(Chola, Fadnes et al. 2015)	1	1	1	1	1	1	1	1	1	1	10
[32]	(Norman, Haas et al. 2009)	1	1	1	1	1	1	1	1	1	1	10
[33]	(Huang, Clermont et al. 2007)	1	1	1	1	1	1	1	1	1	1	10
[34]	(Thokala, Goodacre et al. 2015)	1	1	1	1	1	1	1	1	1	1	10
[35]	(Prinja, Bahuguna et al. 2016)	1	1	1	1	1	1	1	1	1	1	10
[36]	(Zhang, Incardona et al. 2017)	1	1	1	1	1	1	1	0	1	1	9
[37]	(Horton, Gelband et al. 2017)	1	1	1	1	1	1	1	1	1	1	10
[38]	(Fiedler, Mubanga et al. 2014)	1	1	1	1	1	1	1	0	1	1	9
[39]	(Ciaranello, Myer et al. 2015)	1	1	1	1	1	1	1	1	1	1	10
[40]	(Baltussen, Sylla et al. 2005)	1	1	1	1	1	1	1	1	1	1	10
[41]	(Petrou, Boland et al. 2014)	1	1	1	1	1	1	0	1	1	1	9
[42]	(Dayton, Kanter et al. 2006)	1	1	1	1	1	1	1	1	1	1	10
[43]	(Caviness, Cantor et al. 2004)	1	1	1	1	1	1	1	1	1	1	10
[44]	(Sutherland, Downing et al. 2013)	1	1	1	1	1	1	0	1	1	1	9
[45]	(Murray, Lauer et al. 2003)	1	1	1	0	1	1	1	1	1	1	9
[46]	(Xie, da Silva et al. 2012)	1	1	1	1	1	1	0	1	1	1	9
[47]	(Roberts, Arguedas et al. 2007)	1	1	1	1	1	1	1	1	1	1	10
[48]	(Shen, Schneider et al. 2018)	1	1	1	1	1	1	0	1	1	1	9
[49]	(Gerson and Kamal 2008)	1	1	1	1	1	1	0	1	1	0	8
[50]	(van den Brand, Verhave et al. 2017)	1	1	1	1	1	1	1	1	1	0	9
[51]	(Wilson, Jayne et al. 2007)	1	1	1	1	1	1	0	1	1	0	8
[52]	(Motiwala, Gupta et al. 2006)	1	1	1	1	1	1	1	1	1	0	9
[53]	(Perovic and Jankovic 2009)	1	1	1	1	1	1	0	1	0	0	7
[54]	(Tu, Pemberton et al. 2015)	1	1	1	1	1	1	1	1	1	0	9
[55]	(Luca, Burnett et al. 2016)	1	1	1	1	1	1	1	1	1	0	9
[56]	(Pitt, Garside et al. 2006)	1	1	1	1	1	1	0	1	1	0	8
[57]	(Hansen and Chapman 2008)	1	1	1	1	1	1	1	0	1	1	9
[58]	(Basu, Shankar et al. 2016)	1	1	1	1	1	1	1	0	1	0	8
[59]	(Kuznik, Habib et al. 2016)	1	1	1	1	1	1	1	1	1	0	9
[60]	(Mathewos, Owen et al. 2017)	1	1	1	1	1	1	1	1	1	1	10
[61]	(Castro Jaramillo, Moreno Viscaya et al. 2016)	1	1	1	1	1	1	1	1	1	0	9
[62]	(Rae, Furlong et al. 2014)	1	1	1	1	1	1	1	1	1	1	10
[63]	(Simkiss, Snooks et al. 2013)	1	1	1	0	1	1	0	1	1	1	8
[64]	(Gutierrez de Mesa, Hidalgo et al. 2007)	1	1	1	1	1	1	1	1	1	1	10
[65]	(Harris, Washington et al. 2004)	1	1	1	1	1	1	1	1	1	1	10
[66]	(Shaheen, Persson et al. 2015)	1	1	1	1	1	1	1	1	1	1	10

Ref.	Authors, Year	C1	C2	C3	C4	C5	C6	C7	C8	C9	C10	SC
[67]	(Pulkki-Brannstrom, Wolff et al. 2011)	1	1	1	1	1	1	1	1	1	1	10
[68]	(Svefors, Selling et al. 2018)	1	1	1	1	1	1	1	1	1	1	10
[69]	(Baltussen, Knai et al. 2004)	1	1	1	1	1	1	1	1	1	1	10
[70]	(Hutton, Rodriguez et al. 2013)	1	1	1	1	1	1	0	1	1	1	9
[71]	(Fiedler and Chuko 2008)	1	1	1	1	1	1	0	1	1	1	9
[72]	(Koethe, Marseille et al. 2014)	1	1	1	1	1	1	1	1	1	1	10
[73]	(Gulliford, Bhattarai et al. 2014)	1	1	1	1	1	1	1	1	1	1	10
[74]	(Beaston-Blaakman, Shepard et al. 2007)	1	1	1	1	1	1	0	1	1	1	9
[75]	(Yu, Li et al. 2014)	1	1	1	1	1	1	1	1	1	1	10
[76]	(Wilford, Golden et al. 2012)	1	1	0	1	0	0	1	1	1	1	7
[77]	(Sharieff, Horton et al. 2006)	1	1	1	1	1	0	1	1	1	0	8
[78]	(Lividini and Fiedler 2015)	1	1	1	1	1	1	1	1	1	1	10
[79]	(Alonzo González, Menéndez et al. 2000)	1	1	1	1	1	1	1	1	1	1	10
[80]	(Borghi, Guinness et al. 2002)	1	1	0	1	0	1	1	1	0	1	7
[81]	(Eberwein, Kakietek et al. 2016)	1	1	1	1	1		1	1	0	0	8
[82]	(Ekwaru, Ohinmaa et al. 2017)	1	1	1	1	1	1	1	1	1	1	10
[83]	(Graziose, Koch et al. 2017)	1	1	1	1	1	1	1	1	1	1	10
[84]	(Wright, Austin et al. 2014)	1	1	1	1	1	1	1	1	1	1	10
[85]	(Verhaeghe, De Smedt et al. 2014)	1	1	1	1	1	1	1	1	1	1	10
[86]	(Norman, Pirlich et al. 2011)	1	1	1	1	1	1	1	1	1	1	10
[87]	(Webb, Fahimi et al. 2017)	1	1	1	0	1	1	1	1	1	0	8
[88]	(Fitzgerald, Murphy et al. 2018)	1	1	1	1	1	1	1	1	1	0	9
[89]	(Zhong, Cohen et al. 2017)	1	1	1	1	1	1	0	1	1	0	8
[90]	(Akanni, Smith et al. 2017)	1	1	1	1	1	1	0	1	1	0	8
[91]	(Elia, Parsons et al. 2018)	1	1	1	1	1	1	0	1	1	0	8
[92]	(Sharma, Thompson et al. 2018)	1	1	1	1	1	1	0	1	1	0	8
[93]	(Hogan, Baltussen et al. 2005)	1	1	1	1	1	1	1	1	1	1	10
[94]	(Leelukkanaveer, Sithisarankul et al. 2010)	1	1	1	1	1	1	1	1	1	1	10
[95]	(Galarraga, Shah et al. 2018)	1	1	1	1	1	1	0	0	1	1	8
[96]	(Herida, Larsen et al. 2006)	1	1	1	1	1	1	1	1	1	1	10
[97]	(Walensky, Park et al. 2012)	1	1	1	1	1	1	1	1	1	1	10
[98]	(Marshall, Kleinman et al. 2004)	1	1	1	1	1	1	0	1	1	1	9
[99]	(Ramachandran, Manabe et al. 2017)	1	1	1	1	1	1	1	1	1	1	10
[100]	(Gilbert, Shenoi et al. 2016)	1	1	1	1	1	1	1	1	1	1	10
[101]	(Adelman, McFarland et al. 2018)	1	1	1	1	1	1	1	1	1	1	10
[102]	(Uthman and Uthman 2017)	1	1	1	1	1	1	0	1	1	0	8

Ref.	Authors, Year	C1	C2	C3	C4	C5	C6	C7	C8	C9	C10	SC
[103]	(Uhler, Kumarasamy et al. 2010)	1	1	1	1	1	1	1	1	1	1	10
[104]	(Rahman, Khan et al. 2015)	1	1	1	1	1	1	0	0	1	1	8
[105]	(Gutierrez-Delgado, Baez-Mendoza et al. 2008)	1	1	1	1	1	1	1	1	1	1	10
[106]	(Kim, Salomon et al. 2007)	1	1	1	1	1	1	0	1	1	1	9
[107]	(Gilson, Mkanje et al. 1997)	1	1	1	1	1	1	1	1	1	1	10
[108]	(Remme, Feenstra, et al. 2009)	1	1	1	1	1	1	0	0	1	1	8
[109]	(Maheu-Giroux and Castro 2014)	1	1	1	1	1	1	1	0	1	0	8
[110]	(Utzinger, Tozan et al. 2001)	1	1	1	1	1	1	1	0	0	1	8
[111]	(Stuckey, Stevenson et al. 2014)	1	1	1	1	1	1	0	0	1	1	8
[112]	(Phillips, Njau et al. 2015)	1	1	1	1	1	1	1	1	1	1	10
[113]	(Pfeil, Borrmann et al. 2014)	1	1	1	1	1	1	1	1	1	1	10
[114]	(Rakuomi, Okalebo et al. 2017)	1	1	1	1	1	1	1	1	1	1	10
[115]	(Lubell, Riewpaiboon et al. 2011)	1	1	1	1	1	1	1	1	1	1	10
[116]	(Devine, Parmiter et al. 2017)	1	1	1	1	1	1	0	1	1	1	9
[117]	(Stone, Kastner et al. 2016)	1	1	1	1	1	1	1	1	1	1	10
[118]	(Turner, Bettis et al. 2018)	1	1	1	1	1	1	1	1	1	1	10
[119]	(Stromme, Baeroe et al. 2014)	1	1	1	1	1	1	1	1	1	1	10
[120]	(Griekspoor, Sondorp et al. 1999)	1	1	1	1	1	1	0	0	1	1	8
[121]	(Fitzpatrick, Sankara et al. 2017)	1	1	1	1	1	1	1	1	1	1	10
[122]	(Heslin, Patel et al. 2017)	1	1	1	1	1	1	0	1	1	0	8
[123]	(Niens, Zelle et al. 2014)	1	1	1	1	1	1	1	1	1	1	10
[124]	(Zelle, Nyarko et al. 2012)	1	1	1	1	1	1	1	1	1	1	10
[125]	(Forde, Chang et al. 2016)	1	1	1	1	1	1	1	1	1	1	10
[126]	(Gupta, Bansal et al. 2011)	1	1	1	1	1	1	1	1	1	1	10
[127]	(Lee, Sudarshan et al. 2013)	1	1	1	1	1	1	1	1	1	1	10
[128]	(van der Linden, Flach et al. 2016)	1	1	1	1	1	1	1	1	1	1	10
[129]	(Hung, Lai et al. 2016)	1	1	1	1	1	1	1	1	1	1	10
[130]	(Yen, Yen et al. 2009)	1	1	1	1	1	1	1	1	1	1	10
[131]	(Cucchetti, Trevisani et al. 2012)	1	1	1	1	1	1	1	1	1	1	10
[132]	(Sheerin, Green et al. 2004)	1	1	1	1	1	1	1	1	1	1	10
[133]	(Rognoni, Ciani et al. 2017)	1	1	1	1	1	1	1	1	1	1	10
[134]	(Aberg, Maklin et al. 2011)	1	1	1	1	1	1	1	1	1	1	10
[135]	(Miners, Martin et al. 2014)	1	1	1	1	1	1	1	1	1	1	10
[136]	(van Hulst, Hubben et al. 2009)	1	1	1	1	1	1	1	1	1	1	10
[137]	(Hutton, So et al. 2010)	1	1	1	1	1	1	1	1	1	1	10
[138]	(Nayagam, Conteh et al. 2016)	1	1	1	1	1	1	1	1	1	1	10

Ref.	Authors, Year	C1	C2	C3	C4	C5	C6	C7	C8	C9	C10	SC
[139]	(Sutton, Edmunds et al. 2008)	1	1	1	1	1	1	1	0	1	1	9
[140]	(Cillo, Spolverato et al. 2015)	1	1	1	1	1	1	1	0	1	1	9
[141]	(Heijnsdijk, de Carvalho et al. 2015)	1	1	1	1	1	1	1	1	1	1	10
[142]	(Hodges, Lotan et al. 2012)	1	1	1	1	1	1	1	1	1	1	10
[143]	(Parker, Woods et al. 2017)	1	1	1	0	1	1	0	1	1	1	8
[144]	(Renner, Shah et al. 2018)	1	1	1	1	1	1	1	0	1	1	9
[145]	(Soini, Martikainen et al. 2011)	1	1	1	1	1	1	1	1	1	1	10
[146]	(McNeil, Brown et al. 2001)	1	1	1	1	1	1	1	1	1	1	10
[147]	(Gaziano, Steyn et al. 2005)	1	1	1	1	1	1	1	1	1	1	10
[148]	(Gaziano, Bertram et al. 2014)	1	1	1	1	1	1	0	1	1	1	9
[149]	(Jafar, Islam et al. 2011)	1	1	1	1	1	1	1	1	1	1	10
[150]	(Richman, Fairley et al. 2016)	1	1	1	1	1	1	1	1	1	1	10
[151]	(Lamotte, Annemans et al. 2006)	1	1	1	1	1	1	0	1	1	1	9
[152]	(Tolla, Norheim et al. 2016)	1	1	1	1	1	1	1	1	1	1	10
[153]	(Michaud, Abraham et al. 2015)	1	1	1	1	1	1	1	1	1	1	10
[154]	(Vaidya, Severens et al. 2014)	1	1	1	1	1	1	1	1	1	1	10
[155]	(Nam, Briggs et al. 2015)	1	1	1	1	1	1	1	1	1	1	10
[156]	(Osnabrugge, Magnuson et al. 2015)	1	1	1	1	1	1	0	1	1	1	9
[157]	(Davies, Sculpher et al. 2013)	1	1	1	1	1	1	1	1	1	1	10
[158]	(Irlam, Mayosi et al. 2013)	1	1	1	1	1	1	1	1	1	1	10
[159]	(Zachariah and Samnaliev 2015)	1	1	1	1	1	1	1	1	1	1	10
[160]	(Watkins, Mvundura et al. 2015)	1	1	1	1	1	1	0	1	1	1	9
[161]	(McMurray, Trueman et al. 2018)	1	1	1	1	1	1	1	1	1	1	10
[162]	(Cadier, Durand-Zaleski et al. 2016)	1	1	1	1	1	1	1	1	1	1	10
[163]	(Gani, Griffin et al. 2010)	1	1	1	1	1	1	1	1	1	1	10
[164]	(Hertel, Kotchie et al. 2012)	1	1	1	1	1	1	1	1	1	1	10
[165]	(Atsou, Crequit et al. 2016)	1	1	1	1	1	1	1	1	1	1	10
[166]	(Milne, Hockey et al. 2014)	1	1	1	1	1	1	1	1	1	1	10
[167]	(Liu, Li et al. 2013)	1	1	1	1	1	1	1	1	1	1	10
[168]	(Basu, Shankar et al. 2016)	1	1	1	1	1	1	1	1	1	1	10
[169]	(Hakansson, Oguttu et al. 2018)	1	1	1	1	1	1	1	1	1	1	10
[170]	(Vetrini, Kiire et al. 2018)	1	1	1	1	1	1	1	1	1	1	10
[171]	(Group 1998)	1	1	1	1	1	1	1	1	1	1	10
[172]	(Treharne, Liu et al. 2014)	1	1	1	1	1	1	1	1	1	1	10
[173]	(Hoerger, Wittenborn et al. 2010)	1	1	1	1	1	1	0	1	1	1	9
[174]	(Thompson, Bartko-Winters et al. 2013)	1	1	1	1	1	1	1	1	1	1	10

Ref.	Authors, Year	C1	C2	C3	C4	C5	C6	C7	C8	C9	C10	SC
[175]	(Henry, De Brouwer et al. 2015)	1	1	1	1	1	1	0	1	1	1	9
[176]	(Mennini, Russo et al. 2014)	1	1	1	1	1	1	0	1	1	1	9
[177]	(Baltussen, Sylla et al. 2004)	1	1	1	1	1	1	1	1	1	1	10
[178]	(Frick, Riva-Clement et al. 2009)	1	1	1	1	1	1	0	1	1	1	9
[179]	(Baltussen, Naus et al. 2009)	1	1	1	1	1	1	1	1	1	1	10
[180]	(Vaahtoranta-Lehtonen, Tuulonen et al. 2007)	1	1	1	1	1	1	1	1	1	1	10
[181]	(Stein, Kim et al. 2012)	1	1	1	1	1	1	1	1	1	1	10
[182]	(Davies, Barnes et al. 2008)	1	1	1	1	1	1	1	1	1	1	10
[183]	(Ising, Smit et al. 2015)	1	1	1	1	1	1	1	1	1	1	10
[184]	(Patel, McCrone et al. 2013)	1	1	1	1	1	1	0	1	1	1	9
[185]	(Remak, Hutton et al. 2003)	1	1	1	1	1	1	1	1	1	1	10
[186]	(Tanajewski, Franklin et al. 2015)	1	1	1	1	1	1	0	1	1	1	9
[187]	(Yu, Lee et al. 2015)	1	1	1	1	1	1	0	1	1	1	9
[188]	(Heuzenroeder, Donnelly et al. 2004)	1	1	1	1	1	1	1	1	1	1	10
[189]	(Nosyk, Guh et al. 2012)	1	1	1	1	1	1	1	1	1	1	10
[190]	(Taylor, Leonardi-Bee et al. 2011)	1	1	1	1	1	1	1	1	1	1	10
[191]	(Achana, Sutton et al. 2016)	1	1	1	1	1	1	0	1	1	1	9
[192]	(Glotzer, Freedberg et al. 1995)	1	1	1	1	1	1	1	1	1	1	10
[193]	(Murphy, Campbell et al. 2016)	1	1	1	1	1	1	1	1	1	1	10
[194]	(Alkire, Vincent et al. 2012)	1	1	1	1	1	1	1	0	1	1	9
[195]	(Sculpher, Manca et al. 2004)	1	1	1	1	1	1	0	1	1	1	9
[196]	(Hullfish, Trowbridge et al. 2011)	1	1	1	1	1	1	0	1	1	1	9
[197]	(Epiu, Alia et al. 2018)	1	1	1	1	1	1	0	1	1	1	9
[198]	(Chanthavilay, Reinharz et al. 2016)	1	1	1	1	1	1	1	1	1	1	10
[199]	(Havrilesky, Maxwell et al. 2009)	1	1	1	1	1	1	1	1	1	1	10
[200]	(Zowall, Cairns et al. 2008)	1	1	1	1	1	1	0	1	1	1	9
[201]	(Yang, Caughey et al. 2011)	1	1	1	1	1	1	1	1	1	1	10
[202]	(Campos, Maza et al. 2015)	1	1	1	1	1	1	1	1	1	1	10
[203]	(Cowett, Golub et al. 2006)	1	1	1	1	1	1	1	1	1	1	10
[204]	(Roberts, Roberts et al. 2016)	1	1	1	1	1	1	1	1	1	1	10
[205]	(Clegg and Guest 2007)	1	1	1	1	1	1	1	1	1	1	10
[206]	(Cox, Carson et al. 2007)	1	1	1	1	1	1	1	1	1	1	10
[207]	(Puri, Pyrdeck et al. 2012)	1	1	1	1	1	1	1	1	1	1	10
[208]	(Hall, Omer et al. 2016)	1	1	1	1	1	1	1	1	1	1	10
[209]	(Brasel, Borgstrom et al. 1999)	1	1	1	1	1	1	1	1	1	1	10
[210]	(Slover, Hoffman et al. 2009)	1	1	1	1	1	1	1	1	1	1	10

Ref.	Authors, Year	C1	C2	C3	C4	C5	C6	C7	C8	C9	C10	SC
[211]	(Rosas and Feldman 2012)	1	1	1	1	1	1	1	1	1	1	10
[212]	(Nwachukwu, So et al. 2017)	1	1	1	1	1	1	1	1	1	1	10
[213]	(Krishnan, Chatterjee et al. 2014)	1	1	1	1	1	1	1	1	1	1	10
[214]	(Tadisina, Chopra et al. 2014)	1	1	1	1	1	1	1	1	1	1	10
[215]	(Heard, Chaboyer et al. 2017)	1	1	1	1	1	1	1	1	1	1	10
[216]	(Warf, Alkire et al. 2011)	1	1	1	1	1	1	0	1	1	1	9
[217]	(Truzzi, Teich et al. 2018)	1	1	1	1	1	1	1	1	1	1	10
[218]	(Green, Rink et al. 2013)	1	1	1	1	1	1	0	1	1	1	9
[219]	(Bucher, Hall et al. 2011)	1	1	1	1	1	1	1	1	1	1	10
[220]	(Shillcutt, Sanders et al. 2013)	1	1	1	1	1	1	1	1	1	1	10
[221]	(Govindarajan, Naimark et al. 2007)	1	1	1	1	1	1	1	1	1	1	10
[222]	(Hamze, Mengiste et al. 2017)	1	1	1	1	1	1	1	0	1	1	9
[223]	(Kameda, Yamada et al. 2017)	1	1	1	1	1	1	1	1	1	1	10
[224]	(Zendejas, Moriarty et al. 2011)	1	1	1	1	1	1	1	1	1	1	10
[225]	(Zanocco, Heller et al. 2012)	1	1	1	1	1	1	1	1	1	1	10
[226]	(Panca, Viner et al. 2018)	1	1	1	1	1	1	1	1	1	1	10
[227]	(Kent, Korn et al. 2005)	1	1	1	1	1	1	1	1	1	1	10
[228]	(Slover, Espehaug et al. 2006)	1	1	1	1	1	1	1	1	1	1	10
[229]	(Chen, Pedtke et al. 2012)	1	1	1	1	1	1	1	1	1	1	10
[230]	(Taleban, Van Oijen et al. 2016)	1	1	1	1	1	1	1	1	1	1	10
[231]	(Park, Tsai et al. 2012)	1	1	1	1	1	1	1	1	1	1	10
[232]	(Tan, Jacovides et al. 2008)	1	1	1	1	1	1	1	1	1	1	10
[233]	(Hong, Clarke et al. 2016)	1	1	1	1	1	1	1	1	1	1	10
[234]	(Kok, Adang et al. 2007)	1	1	1	1	1	1	1	1	1	1	10
[235]	(Burfeind, Jaik et al. 2010)	1	1	1	1	1	1	0	0	1	1	8
[236]	(Ferguson and Lehman 2003)	1	1	1	1	1	1	1	1	1	1	10
[237]	(Wong and Lang 2014)	1	1	1	1	1	1	1	1	1	1	10
[238]	(Thomas, Tubaro et al. 2015)	1	1	1	1	1	1	1	0	1	1	9
[239]	(Yiee and Baskin 2011)	1	1	1	1	1	1	1	1	1	1	10
[240]	(Kulkarni, Alibhai et al. 2009)	1	1	1	1	1	1	1	1	1	1	10
[241]	(Torquati, Lutfi et al. 2006)	1	1	1	1	1	1	1	1	1	1	10
[242]	(Deppen, Davis et al. 2014)	1	1	1	1	1	1	1	1	1	1	10
[243]	(Morton, Howard et al. 2009)	1	1	1	1	1	1	1	1	1	1	10
[244]	(Javanbakht, Azuara-Blanco et al. 2017)	1	1	1	1	1	1	1	1	1	1	10
[245]	(Leung, Pechlivanoglou et al. 2017)	1	1	1	1	1	1	1	1	1	1	10
[246]	(Hirneiss, Neubauer et al. 2006)	1	1	1	1	1	1	1	1	1	1	10

Ref.	Authors, Year	C1	C2	C3	C4	C5	C6	C7	C8	C9	C10	SC
[247]	(Beauchamp, Beauchamp et al. 2006)	1	1	1	1	1	1	1	1	1	1	10
[248]	(Chang and Smiddy 2014)	1	1	1	1	1	1	1	1	1	1	10
[249]	(Moriarty, Borah et al. 2015)	1	1	1	1	1	1	1	1	1	1	10
[250]	(Busbee, Brown et al. 2003)	1	1	1	1	1	1	1	1	1	1	10
[251]	(Rudmik, Smith et al. 2016)	1	1	1	1	1	1	1	1	1	1	10
[252]	(Rudmik, Soler et al. 2015)	1	1	1	1	1	1	1	1	1	1	10
[253]	(Tseng, Lai et al. 2018)	1	1	1	1	1	1	1	1	1	1	10
[254]	(Ward, Bonomo et al. 2012)	1	1	1	1	1	1	1	1	1	1	10
[255]	(Manchikanti, Falco et al. 2013)	1	1	1	1	1	1	1	0	1	1	9
[256]	(Mwamburi, Tenaglia et al. 2018)	1	1	1	1	1	1	1	1	1	1	10
[257]	(Mohr, Stoltze et al. 2018)	1	1	1	1	1	1	1	1	1	1	10
[258]	(Feller-Kopman, Liu et al. 2017)	1	1	1	1	1	1	1	1	1	1	10
[259]	(Borghi, Guinness et al. 2002)	1	1	1	1	1	1	1	1	1	1	10
[260]	(Shrestha, Marseille et al. 2006)	1	1	1	1	1	1	0	1	1	1	9
[261]	(Manasyan, Chomba et al. 2011)	1	1	1	1	1	1	1	1	1	1	10
[262]	(Dalziel, Segal et al. 2010)	1	1	1	1	1	1	1	1	1	1	10
[263]	(Rahman, Bose et al. 2012)	1	1	1	1	1	1	1	1	1	1	10
[264]	(Bertram, Lim et al. 2010)	1	1	1	1	1	1	1	1	1	1	10
[265]	(Townsend, Greenland et al. 2017)	1	1	1	1	1	1	1	1	1	1	10
[266]	(Rubinstein, Garcia Marti et al. 2009)	1	1	1	1	1	1	1	1	1	1	10
[267]	(Ngalesoni, Ruhago et al. 2017)	1	1	1	1	1	1	1	1	1	1	10
[268]	(Dalziel and Segal 2007)	1	1	1	1	1	1	1	1	1	1	10

Reference

1. Jha P, Bangoura O, Ranson K: **The cost-effectiveness of forty health interventions in Guinea.** *Health Policy Plan* 1998, **13:**249-262.
2. Stover J, Rosen JE, Carvalho MN, Korenromp EL, Friedman HS, Cogan M, Deperthes B: **The case for investing in the male condom.** *PLoS One* 2017, **12:**e0177108.
3. Mvundura M, Nundy N, Kilbourne-Brook M, Coffey PS: **Estimating the hypothetical dual health impact and cost-effectiveness of the Woman's Condom in selected sub-Saharan African countries.** *Int J Womens Health* 2015, **7:**271-277.
4. Sonnenberg FA, Burkman RT, Hagerty CG, Speroff L, Speroff T: **Costs and net health effects of contraceptive methods.** *Contraception* 2004, **69:**447-459.
5. Fragoulakis V, Kourlaba G, Tarlatzis B, Mastrominas M, Maniadakis N: **Economic evaluation of alternative assisted reproduction techniques in management of infertility in Greece.** *Clinicoecon Outcomes Res* 2012, **4:**185-192.

6. Hu D, Grossman D, Levin C, Blanchard K, Goldie SJ: **Cost-effectiveness analysis of alternative first-trimester pregnancy termination strategies in Mexico City.** *BJOG* 2009, **116:**768-779.
7. Garside R, Stein K, Wyatt K, Round A, Pitt M: **A cost-utility analysis of microwave and thermal balloon endometrial ablation techniques for the treatment of heavy menstrual bleeding.** *BJOG* 2004, **111:**1103-1114.
8. Campos NG, Sharma M, Clark A, Lee K, Geng F, Regan C, Kim J, Resch S: **The health and economic impact of scaling cervical cancer prevention in 50 low- and lower-middle-income countries.** *Int J Gynaecol Obstet* 2017, **138 Suppl 1:**47-56.
9. Aledort JE, Hook EW, 3rd, Weinstein MC, Goldie SJ: **The cost effectiveness of gonorrhea screening in urban emergency departments.** *Sex Transm Dis* 2005, **32:**425-436.
10. Looker KJ, Wallace LA, Turner KM: **Impact and cost-effectiveness of chlamydia testing in Scotland: a mathematical modelling study.** *Theor Biol Med Model* 2015, **12:**2.
11. Hansen KS, Chapman G: **Setting priorities for the health care sector in Zimbabwe using cost-effectiveness analysis and estimates of the burden of disease.** *Cost Effectiveness and Resource Allocation* 2008, **6:**14.
12. Eells SJ, Bharadwa K, McKinnell JA, Miller LG: **Recurrent Urinary Tract Infections Among Women: Comparative Effectiveness of 5 Prevention and Management Strategies Using a Markov Chain Monte Carlo Model.** *Clinical Infectious Diseases* 2013, **58:**147-160.
13. Cooper K, Shepherd J, Picot J, Jones J, Kavanagh J, Harden A, Barnett-Page E, Clegg A, Hartwell D, Frampton G, Price A: **An economic model of school-based behavioral interventions to prevent sexually transmitted infections.** *Int J Technol Assess Health Care* 2012, **28:**407-414.
14. Hogan DR, Baltussen R, Hayashi C, Lauer JA, Salomon JA: **Cost effectiveness analysis of strategies to combat HIV/AIDS in developing countries.** *BMJ* 2005.
15. Chola L, McGee S, Tugendhaft A, Buchmann E, Hofman K: **Scaling Up Family Planning to Reduce Maternal and Child Mortality: The Potential Costs and Benefits of Modern Contraceptive Use in South Africa.** *PLOS ONE* 2015, **10:**e0130077.
16. Burgos JL, Gaebler JA, Strathdee SA, Lozada R, Staines H, Patterson TL: **Cost-Effectiveness of an Intervention to Reduce HIV/STI Incidence and Promote Condom Use among Female Sex Workers in the Mexico–US Border Region.** *PLOS ONE* 2010, **5:**e11413.
17. Norman R, Spencer A, Eldridge S, Feder G: **Cost-effectiveness of a programme to detect and provide better care for female victims of intimate partner violence.** *J Health Serv Res Policy* 2010, **15:**143-149.
18. Juillard C, Smith R, Anaya N, Garcia A, Kahn JG, Dicker RA: **Saving lives and saving money: hospital-based violence intervention is cost-effective.** *J Trauma Acute Care Surg* 2015, **78:**252-257; discussion 257-258.
19. Willcox M, Moorthy A, Mohan D, Romano K, Hutchful D, Mehl G, Labrique A, LeFevre A: **Mobile Technology for Community Health in Ghana: Is Maternal Messaging and Provider Use of Technology Cost-Effective in Improving Maternal and Child Health Outcomes at Scale?** *J Med Internet Res* 2019, **21:**e11268.
20. Adam T, Lim SS, Mehta S, Bhutta ZA, Fogstad H, Mathai M, Zupan J, Darmstadt GL: **Cost effectiveness analysis of strategies for maternal and neonatal health in developing countries.** *BMJ* 2005, **331:**1107.
21. Shmueli A, Meiri H, Gonen R: **Economic assessment of screening for pre-eclampsia.** *Prenat Diagn* 2012, **32:**29-38.
22. Lohse N, Marseille E, Kahn JG: **Development of a model to assess the cost-effectiveness of gestational diabetes mellitus screening and lifestyle change for the prevention of type 2 diabetes mellitus.** *Int J Gynaecol Obstet* 2011, **115 Suppl 1:**S20-25.
23. Profit J, Lee D, Zupancic JA, Papile L, Gutierrez C, Goldie SJ, Gonzalez-Pier E, Salomon JA: **Clinical benefits, costs, and cost-effectiveness of neonatal intensive care in Mexico.** *PLoS Med* 2010, **7:**e1000379.
24. Corlew DS, Alkire BC, Poenaru D, Meara JG, Shrime MG: **Economic valuation of the impact of a large surgical charity using the value of lost welfare approach.** *BMJ Glob Health* 2016, **1:**e000059.
25. Cardarelli M, Vaikunth S, Mills K, DiSessa T, Molloy F, Sauter E, Bowtell K, Rivera R, Shin AY, Novick W: **Cost-effectiveness of Humanitarian Pediatric Cardiac Surgery Programs in Low- and Middle-Income Countries.** *JAMA Netw Open* 2018, **1:**e184707.

26. Ochalek J, Revill P, Manthalu G, McGuire F, Nkhoma D, Rollinger A, Sculpher M, Claxton K: **Supporting the development of a health benefits package in Malawi.** *BMJ Glob Health* 2018, **3:**e000607.
27. Antillon M, Bilcke J, Paltiel AD, Pitzer VE: **Cost-effectiveness analysis of typhoid conjugate vaccines in five endemic low- and middle-income settings.** *Vaccine* 2017, **35:**3506-3514.
28. Xie B, da Silva O, Zaric G: **Cost-effectiveness analysis of a system-based approach for managing neonatal jaundice and preventing kernicterus in Ontario.** *Paediatrics & child health* 2012, **17:**11-16.
29. Baltussen R, Smith A: **Cost effectiveness of strategies to combat vision and hearing loss in sub-Saharan Africa and South East Asia: mathematical modelling study.** *BMJ* 2012, **344:**e615.
30. Vossius C, Lotto E, Lyanga S, Mduma E, Msemo G, Perlman J, Ersdal HL: **Cost-effectiveness of the "helping babies breathe" program in a missionary hospital in rural Tanzania.** *PLoS One* 2014, **9:**e102080.
31. Chola L, Fadnes LT, Engebretsen IM, Nkonki L, Nankabirwa V, Sommerfelt H, Tumwine JK, Tylleskar T, Robberstad B, Group P-ES: **Cost-Effectiveness of Peer Counselling for the Promotion of Exclusive Breastfeeding in Uganda.** *PLoS One* 2015, **10:**e0142718.
32. Norman R, Haas M, Chaplin M, Joy P, Wilcken B: **Economic evaluation of tandem mass spectrometry newborn screening in Australia.** *Pediatrics* 2009, **123:**451-457.
33. Huang DT, Clermont G, Dremsizov TT, Angus DC, Pro CI: **Implementation of early goal-directed therapy for severe sepsis and septic shock: A decision analysis.** *Crit Care Med* 2007, **35:**2090-2100.
34. Thokala P, Goodacre S, Ward M, Penn-Ashman J, Perkins GD: **Cost-effectiveness of Out-of-Hospital Continuous Positive Airway Pressure for Acute Respiratory Failure.** *Ann Emerg Med* 2015, **65:**556-563 e556.
35. Prinja S, Bahuguna P, Mohan P, Mazumder S, Taneja S, Bhandari N, van den Hombergh H, Kumar R: **Cost Effectiveness of Implementing Integrated Management of Neonatal and Childhood Illnesses Program in District Faridabad, India.** *PLoS One* 2016, **11:**e0145043.
36. Zhang S, Incardona B, Qazi SA, Stenberg K, Campbell H, Nair H, Severe AWG: **Cost-effectiveness analysis of revised WHO guidelines for management of childhood pneumonia in 74 Countdown countries.** *J Glob Health* 2017, **7:**010409.
37. Horton S, Gelband H, Jamison D, Levin C, Nugent R, Watkins D: **Ranking 93 health interventions for low- and middle-income countries by cost-effectiveness.** *PLoS One* 2017, **12:**e0182951.
38. Fiedler JL, Mubanga F, Siamusantu W, Musonda M, Kabwe KF, Zulu C: **Child Health Week in Zambia: costs, efficiency, coverage and a reassessment of need.** *Health Policy Plan* 2014, **29:**12-29.
39. Ciaranello AL, Myer L, Kelly K, Christensen S, Daskilewicz K, Doherty K, Bekker LG, Hou T, Wood R, Francke JA, et al: **Point-of-care CD4 testing to inform selection of antiretroviral medications in south african antenatal clinics: a cost-effectiveness analysis.** *PLoS One* 2015, **10:**e0117751.
40. Baltussen R, Floyd K, Dye C: **Cost effectiveness analysis of strategies for tuberculosis control in developing countries.** *Bmj* 2005, **331:**1364.
41. Petrou S, Boland A, Khan K, Powell C, Kolamunnage-Dona R, Lowe J, Doull I, Hood K, Williamson P: **Economic evaluation of nebulized magnesium sulphate in acute severe asthma in children.** *Int J Technol Assess Health Care* 2014, **30:**354-360.
42. Dayton JD, Kanter KR, Vincent RN, Mahle WT: **Cost-effectiveness of pediatric heart transplantation.** *J Heart Lung Transplant* 2006, **25:**409-415.
43. Caviness AC, Cantor SB, Allen CH, Ward MA: **A cost-effectiveness analysis of bacterial endocarditis prophylaxis for febrile children who have cardiac lesions and undergo urinary catheterization in the emergency department.** *Pediatrics* 2004, **113:**1291-1296.
44. Sutherland T, Downing J, Miller S, Bishai DM, Butrick E, Fathalla MM, Mourad-Youssif M, Ojengbede O, Nsima D, Kahn JG: **Use of the non-pneumatic anti-shock garment (NASG) for life-threatening obstetric hemorrhage: a cost-effectiveness analysis in Egypt and Nigeria.** *PLoS One* 2013, **8:**e62282.
45. Murray CJ, Lauer JA, Hutubessy RC, Niessen L, Tomijima N, Rodgers A, Lawes CM, Evans DB: **Effectiveness and costs of interventions to lower systolic blood pressure and cholesterol: a global and regional analysis on reduction of cardiovascular-disease risk.** *Lancet* 2003, **361:**717-725.

46. Xie B, da Silva O, Zaric G: **Cost-effectiveness analysis of a system-based approach for managing neonatal jaundice and preventing kernicterus in Ontario.** *Paediatr Child Health* 2012, **17:**11-16.
47. Roberts DN, Arguedas MR, Fallon MB: **Cost-effectiveness of screening for hepatopulmonary syndrome in liver transplant candidates.** *Liver Transpl* 2007, **13:**206-214.
48. Shen NT, Schneider Y, Congly SE, Rosenblatt RE, Namn Y, Fortune BE, Jesudian A, Brown RS, Jr.: **Cost Effectiveness of Early Insertion of Transjugular Intrahepatic Portosystemic Shunts for Recurrent Ascites.** *Clin Gastroenterol Hepatol* 2018, **16:**1503-1510 e1503.
49. Gerson L, Kamal A: **Cost-effectiveness analysis of management strategies for obscure GI bleeding.** *Gastrointest Endosc* 2008, **68:**920-936.
50. van den Brand JA, Verhave JC, Adang EM, Wetzels JF: **Cost-effectiveness of eculizumab treatment after kidney transplantation in patients with atypical haemolytic uraemic syndrome.** *Nephrol Dial Transplant* 2017, **32:**i115-i122.
51. Wilson EC, Jayne DR, Dellow E, Fordham RJ: **The cost-effectiveness of mycophenolate mofetil as firstline therapy in active lupus nephritis.** *Rheumatology (Oxford)* 2007, **46:**1096-1101.
52. Motiwala SS, Gupta S, Lilly MB, Ungar WJ, Coyte PC: **The cost-effectiveness of expanding intensive behavioural intervention to all autistic children in Ontario: in the past year, several court cases have been brought against provincial governments to increase funding for Intensive Behavioural Intervention (IBI). This economic evaluation examines the costs and consequences of expanding an IBI program.** *Healthcare policy = Politiques de sante* 2006, **1:**135-151.
53. Perovic S, Jankovic S: **Renal transplantation vs hemodialysis: cost-effectiveness analysis.** *Vojnosanit Pregl* 2009, **66:**639-644.
54. Tu HY, Pemberton J, Lorenzo AJ, Braga LH: **Economic analysis of continuous antibiotic prophylaxis for prevention of urinary tract infections in infants with high-grade hydronephrosis.** *J Pediatr Urol* 2015, **11:**247 e241-248.
55. Luca NJ, Burnett HF, Ungar WJ, Moretti ME, Beukelman T, Feldman BM, Schwartz G, Bayoumi AM: **Cost-Effectiveness Analysis of First-Line Treatment With Biologic Agents in Polyarticular Juvenile Idiopathic Arthritis.** *Arthritis Care Res (Hoboken)* 2016, **68:**1803-1811.
56. Pitt M, Garside R, Stein K: **A cost-utility analysis of pimecrolimus vs. topical corticosteroids and emollients for the treatment of mild and moderate atopic eczema.** *Br J Dermatol* 2006, **154:**1137-1146.
57. Hansen KS, Chapman G: **Setting priorities for the health care sector in Zimbabwe using cost-effectiveness analysis and estimates of the burden of disease.** *Cost Eff Resour Alloc* 2008, **6:**14.
58. Basu S, Shankar V, Yudkin JS: **Comparative effectiveness and cost-effectiveness of treat-to-target versus benefit-based tailored treatment of type 2 diabetes in low-income and middle-income countries: a modelling analysis.** *The Lancet Diabetes & Endocrinology* 2016, **4:**922-932.
59. Kuznik A, Habib AG, Munube D, Lamorde M: **Newborn screening and prophylactic interventions for sickle cell disease in 47 countries in sub-Saharan Africa: a cost-effectiveness analysis.** *BMC Health Serv Res* 2016, **16:**304.
60. Mathewos B, Owen H, Sitrin D, Cousens S, Degefie T, Wall S, Bekele A, Lawn JE, Daviaud E: **Community-Based Interventions for Newborns in Ethiopia (COMBINE): Cost-effectiveness analysis.** *Health Policy Plan* 2017, **32:**i21-i32.
61. Castro Jaramillo HE, Moreno Viscaya M, Mejia AE: **Cost-Utility Analysis of Primary Prophylaxis, Compared with on-Demand Treatment, for Patients with Severe Hemophilia Type a in Colombia.** *Int J Technol Assess Health Care* 2016, **32:**337-347.
62. Rae C, Furlong W, Jankovic M, Moghrabi A, Naqvi A, Sala A, Samson Y, DePauw S, Feeny D, Barr R: **Economic evaluation of treatment for acute lymphoblastic leukaemia in childhood.** *Eur J Cancer Care (Engl)* 2014, **23:**779-785.
63. Simkiss DE, Snooks HA, Stallard N, Kimani PK, Sewell B, Fitzsimmons D, Anthony R, Winstanley S, Wilson L, Phillips CJ, Stewart-Brown S: **Effectiveness and cost-effectiveness of a universal parenting skills programme in deprived communities: multicentre randomised controlled trial.** *BMJ Open* 2013, **3:**e002851.
64. Gutierrez de Mesa E, Hidalgo I, Christidis P, Ciscar JC, Vegas E, Ibarreta D: **Modeling the impact of genetic screening technologies on healthcare: theoretical model for asthma in children.** *Mol Diagn Ther* 2007, **11:**313-323.
65. Harris RA, Washington AE, Nease RF, Jr., Kuppermann M: **Cost utility of prenatal diagnosis and the risk-based threshold.** *The Lancet* 2004, **363:**276-282.

66. Shaheen R, Persson LA, Ahmed S, Streatfield PK, Lindholm L: **Cost-effectiveness of invitation to food supplementation early in pregnancy combined with multiple micronutrients on infant survival: analysis of data from MINIMat randomized trial, Bangladesh.** *BMC Pregnancy Childbirth* 2015, **15:**125.
67. Pulkki-Brannstrom AM, Wolff C, Brannstrom N, Skordis-Worrall J: **Cost and cost effectiveness of long-lasting insecticide-treated bed nets - a model-based analysis.** *Cost Eff Resour Alloc* 2012, **10:**5.
68. Svefors P, Selling KE, Shaheen R, Khan AI, Persson LA, Lindholm L: **Cost-effectiveness of prenatal food and micronutrient interventions on under-five mortality and stunting: Analysis of data from the MINIMat randomized trial, Bangladesh.** *PLoS One* 2018, **13:**e0191260.
69. Baltussen R, Knai C, Sharan M: **Iron fortification and iron supplementation are cost-effective interventions to reduce iron deficiency in four subregions of the world.** *J Nutr* 2004, **134:**2678-2684.
70. Hutton G, Rodriguez UP, Winara A, Anh NV, Phyrum K, Chuan L, Blackett I, Weitz A: **Economic efficiency of sanitation interventions in Southeast Asia.** *Journal of Water, Sanitation and Hygiene for Development* 2013, **4:**23-36.
71. Fiedler JL, Chuko T: **The cost of Child Health Days: a case study of Ethiopia's Enhanced Outreach Strategy (EOS).** *Health Policy Plan* 2008, **23:**222-233.
72. Koethe JR, Marseille E, Giganti MJ, Chi BH, Heimburger D, Stringer JS: **Estimating the cost-effectiveness of nutrition supplementation for malnourished, HIV-infected adults starting antiretroviral therapy in a resource-constrained setting.** *Cost Eff Resour Alloc* 2014, **12:**10.
73. Gulliford MC, Bhattarai N, Charlton J, Rudisill C: **Cost-effectiveness of a universal strategy of brief dietary intervention for primary prevention in primary care: population-based cohort study and Markov model.** *Cost Eff Resour Alloc* 2014, **12:**4.
74. Beaston-Blaakman A, Shepard DS, Stone N, Shevitz AH: **Cost-effectiveness of clinical interventions for AIDS wasting.** *AIDS Care* 2007, **19:**996-1001.
75. Yu W, Li C, Fu X, Cui Z, Liu X, Fan L, Zhang G, Ma J: **The cost-effectiveness of different feeding patterns combined with prompt treatments for preventing mother-to-child HIV transmission in South Africa: estimates from simulation modeling.** *PLoS One* 2014, **9:**e102872.
76. Wilford R, Golden K, Walker DG: **Cost-effectiveness of community-based management of acute malnutrition in Malawi.** *Health Policy Plan* 2012, **27:**127-137.
77. Sharieff W, Horton SE, Zlotkin S: **Economic gains of a home fortification program: evaluation of "Sprinkles" from the provider's perspective.** *Can J Public Health* 2006, **97:**20-23.
78. Lividini K, Fiedler JL: **Assessing the promise of biofortification: A case study of high provitamin A maize in Zambia.** *Food Policy* 2015, **54:**65-77.
79. Alonzo González M, Menéndez C, Font F, Kahigwa E, Kimario J, Mshinda H, Tanner M, Bosch-Capblanch X, Alonso PL: **Cost-effectiveness of iron supplementation and malaria chemoprophylaxis in the prevention of anaemia and malaria among Tanzanian infants.** *Bulletin of the World Health Organization* 2000, **78:**97-107.
80. Borghi J, Guinness L, Ouedraogo J, Curtis V: **Is hygiene promotion cost-effective? A case study in Burkina Faso.** *Tropical Medicine & International Health* 2002, **7:**960-969.
81. Eberwein JD, Kakietek J, de Beni D, Moloney G, Pereira A, Akuoku JK, Volege M, Matu S, Shekar M: *An Investment Framework for Nutrition in Kenya Reducing Stunting and Other Forms of Child Malnutrition.* World Bank; 2016.
82. Ekwaru JP, Ohinmaa A, Tran BX, Setayeshgar S, Johnson JA, Veugelers PJ: **Cost-effectiveness of a school-based health promotion program in Canada: A life-course modeling approach.** *PLoS One* 2017, **12:**e0177848.
83. Graziose MM, Koch PA, Wang YC, Lee Gray H, Contento IR: **Cost-effectiveness of a Nutrition Education Curriculum Intervention in Elementary Schools.** *J Nutr Educ Behav* 2017, **49:**684-691 e681.
84. Wright DR, Austin SB, LeAnn Noh H, Jiang Y, Sonneville KR: **The cost-effectiveness of school-based eating disorder screening.** *Am J Public Health* 2014, **104:**1774-1782.
85. Verhaeghe N, De Smedt D, De Maeseneer J, Maes L, Van Heeringen C, Annemans L: **Cost-effectiveness of health promotion targeting physical activity and healthy eating in mental health care.** *BMC Public Health* 2014, **14:**856.
86. Norman K, Pirlich M, Smoliner C, Kilbert A, Schulzke JD, Ockenga J, Lochs H, Reinhold T: **Cost-effectiveness of a 3-month intervention with oral nutritional supplements in disease-related malnutrition: a randomised controlled pilot study.** *Eur J Clin Nutr* 2011, **65:**735-742.

87. Webb M, Fahimi S, Singh GM, Khatibzadeh S, Micha R, Powles J, Mozaffarian D: **Cost effectiveness of a government supported policy strategy to decrease sodium intake: global analysis across 183 nations.** *BMJ* 2017, **356:**i6699.
88. Fitzgerald S, Murphy A, Kirby A, Geaney F, Perry IJ: **Cost-effectiveness of a complex workplace dietary intervention: an economic evaluation of the Food Choice at Work study.** *BMJ Open* 2018, **8:**e019182.
89. Zhong Y, Cohen JT, Goates S, Luo M, Nelson J, Neumann PJ: **The Cost-Effectiveness of Oral Nutrition Supplementation for Malnourished Older Hospital Patients.** *Appl Health Econ Health Policy* 2017, **15:**75-83.
90. Akanni OO, Smith ML, Ory MG: **Cost-Effectiveness of a Community Exercise and Nutrition Program for Older Adults: Texercise Select.** *Int J Environ Res Public Health* 2017, **14**.
91. Elia M, Parsons EL, Cawood AL, Smith TR, Stratton RJ: **Cost-effectiveness of oral nutritional supplements in older malnourished care home residents.** *Clin Nutr* 2018, **37:**651-658.
92. Sharma Y, Thompson C, Miller M, Shahi R, Hakendorf P, Horwood C, Kaambwa B: **Economic evaluation of an extended nutritional intervention in older Australian hospitalized patients: a randomized controlled trial.** *BMC Geriatr* 2018, **18:**41.
93. Hogan DR, Baltussen R, Hayashi C, Lauer JA, Salomon JA: **Cost effectiveness analysis of strategies to combat HIV/AIDS in developing countries.** *BMJ* 2005, **331:**1431-1437.
94. Leelukkanaveer Y, Sithisarankul P, Hirunsutthikul N: **Provider-initiated HIV counseling and testing of out patients at community hospitals in Thailand: an economic evaluation using the Markov model.** 2010, **4:**479.
95. Galarraga O, Shah P, Wilson-Barthes M, Ayuku D, Braitstein P: **Cost and cost-effectiveness of voluntary medical male circumcision in street-connected youth: findings from an education-based pilot intervention in Eldoret, Kenya.** *AIDS Res Ther* 2018, **15:**24.
96. Herida M, Larsen C, Lot F, Laporte A, Desenclos JC, Hamers FF: **Cost-effectiveness of HIV post-exposure prophylaxis in France.** *AIDS* 2006, **20:**1753-1761.
97. Walensky RP, Park JE, Wood R, Freedberg KA, Scott CA, Bekker LG, Losina E, Mayer KH, Seage GR, 3rd, Paltiel AD: **The cost-effectiveness of pre-exposure prophylaxis for HIV infection in South African women.** *Clin Infect Dis* 2012, **54:**1504-1513.
98. Marshall DA, Kleinman SH, Wong JB, AuBuchon JP, Grima DT, Kulin NA, Weinstein MC: **Cost-effectiveness of nucleic acid test screening of volunteer blood donations for hepatitis B, hepatitis C and human immunodeficiency virus in the United States.** *Vox Sang* 2004, **86:**28-40.
99. Ramachandran A, Manabe Y, Rajasingham R, Shah M: **Cost-effectiveness of CRAG-LFA screening for cryptococcal meningitis among people living with HIV in Uganda.** *BMC Infect Dis* 2017, **17:**225.
100. Gilbert JA, Shenoi SV, Moll AP, Friedland GH, Paltiel AD, Galvani AP: **Cost-Effectiveness of Community-Based TB/HIV Screening and Linkage to Care in Rural South Africa.** *PLoS One* 2016, **11:**e0165614.
101. Adelman MW, McFarland DA, Tsegaye M, Aseffa A, Kempker RR, Blumberg HM: **Cost-effectiveness of WHO-Recommended Algorithms for TB Case Finding at Ethiopian HIV Clinics.** *Open Forum Infect Dis* 2018, **5:**ofx269.
102. Uthman R, Uthman O: **Cost-effectiveness of early versus delayed antiretroviral therapy in tuberculosis patients infected with HIV in sub-Saharan Africa [version 1; peer review: 2 not approved].** *F1000Research* 2017, **6**.
103. Uhler LM, Kumarasamy N, Mayer KH, Saxena A, Losina E, Muniyandi M, Stoler AW, Lu Z, Walensky RP, Flanigan TP, et al: **Cost-Effectiveness of HIV Testing Referral Strategies among Tuberculosis Patients in India.** *PLOS ONE* 2010, **5:**e12747.
104. Rahman MM, Khan M, Gruber D: **A Low-Cost Partner Notification Strategy for the Control of Sexually Transmitted Diseases: A Case Study From Louisiana.** *Am J Public Health* 2015, **105:**1675-1680.
105. Gutierrez-Delgado C, Baez-Mendoza C, Gonzalez-Pier E, de la Rosa AP, Witlen R: **[Generalized cost-effectiveness of preventive interventions against cervical cancer in Mexican women: results of a Markov model from the public sector perspective].** *Salud Publica Mex* 2008, **50:**107-118.

106. Kim S-Y, Salomon JA, Goldie SJ: **Economic evaluation of hepatitis B vaccination in low-income countries: using cost-effectiveness affordability curves.** *Bulletin of the World Health Organization* 2007, **85:**833-842.
107. Gilson L, Mkanje R, Grosskurth H, Mosha F, Picard J, Gavyole A, Todd J, Mayaud P, Swai R, Fransen L, et al: **Cost-effectiveness of improved treatment services for sexually transmitted diseases in preventing HIV-1 infection in Mwanza Region, Tanzania.** *The Lancet* 1997, **350:**1805-1809.
108. Remme JHF, Feenstra P, Lever PR, Medici AC, Morel CM, Noma M, Ramaiah KD, Richards F, Seketeli A, Schmunis G, et al: **Tropical Diseases Targeted for Elimination: Chagas Disease, Lymphatic Filariasis, Onchocerciasis, and Leprosy.** In *Disease Control Priorities in Developing Countries.* Edited by nd, Jamison DT, Breman JG, Measham AR, Alleyne G, Claeson M, Evans DB, Jha P, Mills A, Musgrove P. Washington (DC); 2006
109. Maheu-Giroux M, Castro MC: **Cost-effectiveness of larviciding for urban malaria control in Tanzania.** *Malar J* 2014, **13:**477.
110. Utzinger J, Tozan Y, Singer BH: **Efficacy and cost-effectiveness of environmental management for malaria control.** *Tropical Medicine & International Health* 2001, **6:**677-687.
111. Stuckey EM, Stevenson J, Galactionova K, Baidjoe AY, Bousema T, Odongo W, Kariuki S, Drakeley C, Smith TA, Cox J, Chitnis N: **Modeling the cost effectiveness of malaria control interventions in the highlands of western Kenya.** *PLoS One* 2014, **9:**e107700.
112. Phillips V, Njau J, Li S, Kachur P: **Simulations Show Diagnostic Testing For Malaria In Young African Children Can Be Cost-Saving Or Cost-Effective.** *Health Aff (Millwood)* 2015, **34:**1196-1203.
113. Pfeil J, Borrmann S, Tozan Y: **Dihydroartemisinin-piperaquine vs. artemether-lumefantrine for first-line treatment of uncomplicated malaria in African children: a cost-effectiveness analysis.** *PLoS One* 2014, **9:**e95681.
114. Rakuomi V, Okalebo F, Ndwigah S, Mbugua L: **Cost effectiveness of pre-referral antimalarial treatment in severe malaria among children in sub-Saharan Africa.** *Cost Eff Resour Alloc* 2017, **15:**14.
115. Lubell Y, Riewpaiboon A, Dondorp AM, von Seidlein L, Mokuolu OA, Nansumba M, Gesase S, Kent A, Mtove G, Olaosebikan R, et al: **Cost-effectiveness of parenteral artesunate for treating children with severe malaria in sub-Saharan Africa.** *Bull World Health Organ* 2011, **89:**504-512.
116. Devine A, Parmiter M, Chu CS, Bancone G, Nosten F, Price RN, Lubell Y, Yeung S: **Using G6PD tests to enable the safe treatment of Plasmodium vivax infections with primaquine on the Thailand-Myanmar border: A cost-effectiveness analysis.** *PLoS Negl Trop Dis* 2017, **11:**e0005602.
117. Stone CM, Kastner R, Steinmann P, Chitnis N, Tanner M, Tediosi F: **Modelling the health impact and cost-effectiveness of lymphatic filariasis eradication under varying levels of mass drug administration scale-up and geographic coverage.** *BMJ Glob Health* 2016, **1:**e000021.
118. Turner HC, Bettis AA, Chu BK, McFarland DA, Hooper PJ, Mante SD, Fitzpatrick C, Bradley MH: **Investment Success in Public Health: An Analysis of the Cost-Effectiveness and Cost-Benefit of the Global Programme to Eliminate Lymphatic Filariasis.** *Clin Infect Dis* 2017, **64:**728-735.
119. Stromme EM, Baeroe K, Norheim OF: **Disease control priorities for neglected tropical diseases: lessons from priority ranking based on the quality of evidence, cost effectiveness, severity of disease, catastrophic health expenditures, and loss of productivity.** *Dev World Bioeth* 2014, **14:**132-141.
120. Griekspoor A, Sondorp E, Vos T: **Cost-effectiveness analysis of humanitarian relief interventions: visceral leishmaniasis treatment in the Sudan.** *Health Policy Plan* 1999, **14:**70-76.
121. Fitzpatrick C, Sankara DP, Agua JF, Jonnalagedda L, Rumi F, Weiss A, Braden M, Ruiz-Tiben E, Kruse N, Braband K, Biswas G: **The cost-effectiveness of an eradication programme in the end game: Evidence from guinea worm disease.** *PLoS Negl Trop Dis* 2017, **11:**e0005922.
122. Heslin M, Patel A, Stahl D, Gardner-Sood P, Mushore M, Smith S, Greenwood K, Onagbesan O, O'Brien C, Fung C, et al: **Randomised controlled trial to improve health and reduce substance use in established psychosis (IMPaCT): cost-effectiveness of integrated psychosocial health promotion.** *BMC Psychiatry* 2017, **17:**407.
123. Niens LM, Zelle SG, Gutierrez-Delgado C, Rivera Pena G, Hidalgo Balarezo BR, Rodriguez Steller E, Rutten FF: **Cost-effectiveness of breast cancer control strategies in Central America: the cases of Costa Rica and Mexico.** *PLoS One* 2014, **9:**e95836.

124. Zelle SG, Nyarko KM, Bosu WK, Aikins M, Niens LM, Lauer JA, Sepulveda CR, Hontelez JA, Baltussen R: **Costs, effects and cost-effectiveness of breast cancer control in Ghana.** *Trop Med Int Health* 2012, **17:**1031-1043.
125. Forde GK, Chang J, Ziogas A: **Cost-effectiveness of primary debulking surgery when compared to neoadjuvant chemotherapy in the management of stage IIIC and IV epithelial ovarian cancer.** *Clinicoecon Outcomes Res* 2016, **8:**397-406.
126. Gupta N, Bansal A, Wani SB, Gaddam S, Rastogi A, Sharma P: **Endoscopy for upper GI cancer screening in the general population: a cost-utility analysis.** *Gastrointest Endosc* 2011, **74:**610-624 e612.
127. Lee L, Sudarshan M, Li C, Latimer E, Fried GM, Mulder DS, Feldman LS, Ferri LE: **Cost-effectiveness of minimally invasive versus open esophagectomy for esophageal cancer.** *Ann Surg Oncol* 2013, **20:**3732-3739.
128. van der Linden N, Flach GB, de Bree R, Uyl-de Groot CA: **Cost-utility of sentinel lymph node biopsy in cT1-T2N0 oral cancer.** *Oral Oncol* 2016, **53:**20-26.
129. Hung MC, Lai WW, Chen HH, Lee JC, Lin YJ, Hsiao JR, Cheng YM, Shan YS, Su WC, Wang JD: **Cost effectiveness of cancer treatment in Taiwan.** *J Formos Med Assoc* 2016, **115:**609-618.
130. Yen RF, Yen MF, Hong RL, Tzen KY, Chien CR, Chen TH: **The cost-utility analysis of 18-fluoro-2-deoxyglucose positron emission tomography in the diagnosis of recurrent nasopharyngeal carcinoma.** *Acad Radiol* 2009, **16:**54-60.
131. Cucchetti A, Trevisani F, Cescon M, Ercolani G, Farinati F, Poggio PD, Rapaccini G, Nolfo MA, Benvegnu L, Zoli M, et al: **Cost-effectiveness of semi-annual surveillance for hepatocellular carcinoma in cirrhotic patients of the Italian Liver Cancer population.** *J Hepatol* 2012, **56:**1089-1096.
132. Sheerin IG, Green FT, Sellman JD: **What is the cost-effectiveness of hepatitis C treatment for injecting drug users on methadone maintenance in New Zealand?** *Drug Alcohol Rev* 2004, **23:**261-272.
133. Rognoni C, Ciani O, Sommariva S, Tarricone R: **Real-World Data for the Evaluation of Transarterial Radioembolization versus Sorafenib in Hepatocellular Carcinoma: A Cost-Effectiveness Analysis.** *Value Health* 2017, **20:**336-344.
134. Aberg F, Maklin S, Rasanen P, Roine RP, Sintonen H, Koivusalo AM, Hockerstedt K, Isoniemi H: **Cost of a quality-adjusted life year in liver transplantation: the influence of the indication and the model for end-stage liver disease score.** *Liver Transpl* 2011, **17:**1333-1343.
135. Miners AH, Martin NK, Ghosh A, Hickman M, Vickerman P: **Assessing the cost-effectiveness of finding cases of hepatitis C infection in UK migrant populations and the value of further research.** *J Viral Hepat* 2014, **21:**616-623.
136. van Hulst M, Hubben GA, Sagoe KW, Promwong C, Permpikul P, Fongsatitkul L, Glynn DM, Sibinga CT, Postma MJ: **Web interface-supported transmission risk assessment and cost-effectiveness analysis of postdonation screening: a global model applied to Ghana, Thailand, and the Netherlands.** *Transfusion* 2009, **49:**2729-2742.
137. Hutton DW, So SK, Brandeau ML: **Cost-effectiveness of nationwide hepatitis B catch-up vaccination among children and adolescents in China.** *Hepatology* 2010, **51:**405-414.
138. Nayagam S, Conteh L, Sicuri E, Shimakawa Y, Suso P, Tamba S, Njie R, Njai H, Lemoine M, Hallett TB, Thursz M: **Cost-effectiveness of community-based screening and treatment for chronic hepatitis B in The Gambia: an economic modelling analysis.** *Lancet Glob Health* 2016, **4:**e568-578.
139. Sutton AJ, Edmunds WJ, Sweeting MJ, Gill ON: **The cost-effectiveness of screening and treatment for hepatitis C in prisons in England and Wales: a cost-utility analysis.** *J Viral Hepat* 2008, **15:**797-808.
140. Cillo U, Spolverato G, Vitale A, Ejaz A, Lonardi S, Cosgrove D, Pawlik TM: **Liver Resection for Advanced Intrahepatic Cholangiocarcinoma: A Cost-Utility Analysis.** *World J Surg* 2015, **39:**2500-2509.
141. Heijnsdijk EA, de Carvalho TM, Auvinen A, Zappa M, Nelen V, Kwiatkowski M, Villers A, Paez A, Moss SM, Tammela TL, et al: **Cost-effectiveness of prostate cancer screening: a simulation study based on ERSPC data.** *J Natl Cancer Inst* 2015, **107:**366.
142. Hodges JC, Lotan Y, Boike TP, Benton R, Barrier A, Timmerman RD: **Cost-effectiveness analysis of stereotactic body radiation therapy versus intensity-modulated radiation therapy: an emerging initial radiation treatment option for organ-confined prostate cancer.** *J Oncol Pract* 2012, **8:**e31s-37s.

143. Parker C, Woods B, Eaton J, Ma E, Selby R, Benson E, Engstrom A, Sajosi P, Briggs A, Bonthapally V: **Brentuximab vedotin in relapsed/refractory Hodgkin lymphoma post-autologous stem cell transplant: a cost-effectiveness analysis in Scotland.** *J Med Econ* 2017, **20:**8-18.
144. Renner L, Shah S, Bhakta N, Denburg A, Horton S, Gupta S: **Evidence From Ghana Indicates That Childhood Cancer Treatment in Sub-Saharan Africa Is Very Cost Effective: A Report From the Childhood Cancer 2030 Network.** *J Glob Oncol* 2018, **4:**1-9.
145. Soini EJ, Martikainen JA, Nousiainen T: **Treatment of follicular non-Hodgkin's lymphoma with or without rituximab: cost-effectiveness and value of information based on a 5-year follow-up.** *Ann Oncol* 2011, **22:**1189-1197.
146. McNeil DE, Brown M, Ching A, DeBaun MR: **Screening for Wilms tumor and hepatoblastoma in children with Beckwith-Wiedemann syndromes: a cost-effective model.** *Med Pediatr Oncol* 2001, **37:**349-356.
147. Gaziano TA, Steyn K, Cohen DJ, Weinstein MC, Opie LH: **Cost-effectiveness analysis of hypertension guidelines in South Africa: absolute risk versus blood pressure level.** *Circulation* 2005, **112:**3569-3576.
148. Gaziano TA, Bertram M, Tollman SM, Hofman KJ: **Hypertension education and adherence in South Africa: a cost-effectiveness analysis of community health workers.** *BMC Public Health* 2014, **14:**240.
149. Jafar TH, Islam M, Bux R, Poulter N, Hatcher J, Chaturvedi N, Ebrahim S, Cosgrove P, Hypertension Research G: **Cost-effectiveness of community-based strategies for blood pressure control in a low-income developing country: findings from a cluster-randomized, factorial-controlled trial.** *Circulation* 2011, **124:**1615-1625.
150. Richman IB, Fairley M, Jorgensen ME, Schuler A, Owens DK, Goldhaber-Fiebert JD: **Cost-effectiveness of Intensive Blood Pressure Management.** *JAMA Cardiol* 2016, **1:**872-879.
151. Lamotte M, Annemans L, Evers T, Kubin M: **A multi-country economic evaluation of low-dose aspirin in the primary prevention of cardiovascular disease.** *Pharmacoeconomics* 2006, **24:**155-169.
152. Tolla MT, Norheim OF, Memirie ST, Abdisa SG, Ababulgu A, Jerene D, Bertram M, Strand K, Verguet S, Johansson KA: **Prevention and treatment of cardiovascular disease in Ethiopia: a cost-effectiveness analysis.** *Cost Eff Resour Alloc* 2016, **14:**10.
153. Michaud TL, Abraham J, Jalal H, Luepker RV, Duval S, Hirsch AT: **Cost-Effectiveness of a Statewide Campaign to Promote Aspirin Use for Primary Prevention of Cardiovascular Disease.** *J Am Heart Assoc* 2015, **4**.
154. Vaidya A, Severens JL, Bongaerts BW, Cleutjens KB, Nelemans PJ, Hofstra L, van Dieijen-Visser M, Biessen EA: **High-sensitive troponin T assay for the diagnosis of acute myocardial infarction: an economic evaluation.** *BMC Cardiovasc Disord* 2014, **14:**77.
155. Nam J, Briggs A, Layland J, Oldroyd KG, Curzen N, Sood A, Balachandran K, Das R, Junejo S, Eteiba H, et al: **Fractional flow reserve (FFR) versus angiography in guiding management to optimise outcomes in non-ST segment elevation myocardial infarction (FAMOUS-NSTEMI) developmental trial: cost-effectiveness using a mixed trial- and model-based methods.** *Cost Eff Resour Alloc* 2015, **13:**19.
156. Osnabrugge RL, Magnuson EA, Serruys PW, Campos CM, Wang K, van Klaveren D, Farooq V, Abdallah MS, Li H, Vilain KA, et al: **Cost-effectiveness of percutaneous coronary intervention versus bypass surgery from a Dutch perspective.** *Heart* 2015, **101:**1980-1988.
157. Davies A, Sculpher M, Barrett A, Huete T, Sacristan JA, Dilla T: **Prasugrel compared to clopidogrel in patients with acute coronary syndrome undergoing percutenaous coronary intervention: a Spanish model-based cost effectiveness analysis.** *Farm Hosp* 2013, **37:**307-316.
158. Irlam J, Mayosi BM, Engel M, Gaziano TA: **Primary prevention of acute rheumatic fever and rheumatic heart disease with penicillin in South African children with pharyngitis: a cost-effectiveness analysis.** *Circ Cardiovasc Qual Outcomes* 2013, **6:**343-351.
159. Zachariah JP, Samnaliev M: **Echo-based screening of rheumatic heart disease in children: a cost-effectiveness Markov model.** *J Med Econ* 2015, **18:**410-419.
160. Watkins DA, Mvundura M, Nordet P, Mayosi BM: **A cost-effectiveness analysis of a program to control rheumatic fever and rheumatic heart disease in Pinar del Rio, Cuba.** *PLoS One* 2015, **10:**e0121363.
161. McMurray JJV, Trueman D, Hancock E, Cowie MR, Briggs A, Taylor M, Mumby-Croft J, Woodcock F, Lacey M, Haroun R, Deschaseaux C: **Cost-effectiveness of sacubitril/valsartan in the treatment of heart failure with reduced ejection fraction.** *Heart* 2018, **104:**1006-1013.

162. Cadier B, Durand-Zaleski I, Thomas D, Chevreul K: **Cost Effectiveness of Free Access to Smoking Cessation Treatment in France Considering the Economic Burden of Smoking-Related Diseases.** *PLoS One* 2016, **11:**e0148750.
163. Gani R, Griffin J, Kelly S, Rutten-van Molken M: **Economic analyses comparing tiotropium with ipratropium or salmeterol in UK patients with COPD.** *Prim Care Respir J* 2010, **19:**68-74.
164. Hertel N, Kotchie RW, Samyshkin Y, Radford M, Humphreys S, Jameson K: **Cost-effectiveness of available treatment options for patients suffering from severe COPD in the UK: a fully incremental analysis.** *Int J Chron Obstruct Pulmon Dis* 2012, **7:**183-199.
165. Atsou K, Crequit P, Chouaid C, Hejblum G: **Simulation-Based Estimates of the Effectiveness and Cost-Effectiveness of Pulmonary Rehabilitation in Patients with Chronic Obstructive Pulmonary Disease in France.** *PLoS One* 2016, **11:**e0156514.
166. Milne RJ, Hockey H, Rea H: **Long-term air humidification therapy is cost-effective for patients with moderate or severe chronic obstructive pulmonary disease or bronchiectasis.** *Value Health* 2014, **17:**320-327.
167. Liu X, Li C, Gong H, Cui Z, Fan L, Yu W, Zhang C, Ma J: **An economic evaluation for prevention of diabetes mellitus in a developing country: a modelling study.** *BMC Public Health* 2013, **13:**729.
168. Basu S, Shankar V, Yudkin JS: **Comparative effectiveness and cost-effectiveness of treat-to-target versus benefit-based tailored treatment of type 2 diabetes in low-income and middle-income countries: a modelling analysis.** *Lancet Diabetes Endocrinol* 2016, **4:**922-932.
169. Hakansson M, Oguttu M, Gemzell-Danielsson K, Makenzius M: **Human rights versus societal norms: a mixed methods study among healthcare providers on social stigma related to adolescent abortion and contraceptive use in Kisumu, Kenya.** *BMJ Glob Health* 2018, **3:**e000608.
170. Vetrini D, Kiire CA, Burgess PI, Harding SP, Kayange PC, Kalua K, Msukwa G, Beare NAV, Madan J: **Incremental cost-effectiveness of screening and laser treatment for diabetic retinopathy and macular edema in Malawi.** *PLoS One* 2018, **13:**e0190742.
171. Group TCDC-ES: **The Cost-effectiveness of Screening for Type 2 Diabetes.** *JAMA* 1998, **280:**1757-1763.
172. Treharne C, Liu FX, Arici M, Crowe L, Farooqui U: **Peritoneal dialysis and in-centre haemodialysis: a cost-utility analysis from a UK payer perspective.** *Appl Health Econ Health Policy* 2014, **12:**409-420.
173. Hoerger TJ, Wittenborn JS, Segel JE, Burrows NR, Imai K, Eggers P, Pavkov ME, Jordan R, Hailpern SM, Schoolwerth AC, et al: **A health policy model of CKD: 2. The cost-effectiveness of microalbuminuria screening.** *Am J Kidney Dis* 2010, **55:**463-473.
174. Thompson M, Bartko-Winters S, Bernard L, Fenton A, Hutchison C, Di Iorio B: **Economic evaluation of sevelamer for the treatment of hyperphosphatemia in chronic kidney disease patients not on dialysis in the United Kingdom.** *J Med Econ* 2013, **16:**744-755.
175. Henry TL, De Brouwer BF, Van Keep MM, Blankestijn PJ, Bots ML, Koffijberg H: **Cost-effectiveness of renal denervation therapy for the treatment of resistant hypertension in The Netherlands.** *J Med Econ* 2015, **18:**76-87.
176. Mennini FS, Russo S, Marcellusi A, Quintaliani G, Fouque D: **Economic effects of treatment of chronic kidney disease with low-protein diet.** *J Ren Nutr* 2014, **24:**313-321.
177. Baltussen R, Sylla M, Mariotti SP: **Cost-effectiveness analysis of cataract surgery: a global and regional analysis.** *Bulletin of the World Health Organization* 2004, **82:**338-345.
178. Frick KD, Riva-Clement L, Shankar MB: **Screening for refractive error and fitting with spectacles in rural and urban India: cost-effectiveness.** *Ophthalmic Epidemiol* 2009, **16:**378-387.
179. Baltussen R, Naus J, Limburg H: **Cost-effectiveness of screening and correcting refractive errors in school children in Africa, Asia, America and Europe.** *Health Policy* 2009, **89:**201-215.
180. Vaahtoranta-Lehtonen H, Tuulonen A, Aronen P, Sintonen H, Suoranta L, Kovanen N, Linna M, Laara E, Malmivaara A: **Cost effectiveness and cost utility of an organized screening programme for glaucoma.** *Acta Ophthalmol Scand* 2007, **85:**508-518.

181. Stein JD, Kim DD, Peck WW, Giannetti SM, Hutton DW: **Cost-effectiveness of medications compared with laser trabeculoplasty in patients with newly diagnosed open-angle glaucoma.** *Arch Ophthalmol* 2012, **130:**497-505.
182. Davies LM, Barnes TR, Jones PB, Lewis S, Gaughran F, Hayhurst K, Markwick A, Lloyd H, Team C: **A randomized controlled trial of the cost-utility of second-generation antipsychotics in people with psychosis and eligible for clozapine.** *Value Health* 2008, **11:**549-562.
183. Ising HK, Smit F, Veling W, Rietdijk J, Dragt S, Klaassen RM, Savelsberg NS, Boonstra N, Nieman DH, Linszen DH, et al: **Cost-effectiveness of preventing first-episode psychosis in ultra-high-risk subjects: multi-centre randomized controlled trial.** *Psychol Med* 2015, **45:**1435-1446.
184. Patel A, McCrone P, Leese M, Amaddeo F, Tansella M, Kilian R, Angermeyer M, Kikkert M, Schene A, Knapp M: **Cost-effectiveness of adherence therapy versus health education for people with schizophrenia: randomised controlled trial in four European countries.** *Cost Eff Resour Alloc* 2013, **11:**12.
185. Remak E, Hutton J, Price M, Peeters K, Adriaenssen I: **A Markov model of treatment of newly diagnosed epilepsy in the UK. An initial assessment of cost-effectiveness of topiramate.** *Eur J Health Econ* 2003, **4:**271-278.
186. Tanajewski L, Franklin M, Gkountouras G, Berdunov V, Harwood RH, Goldberg SE, Bradshaw LE, Gladman JR, Elliott RA: **Economic Evaluation of a General Hospital Unit for Older People with Delirium and Dementia (TEAM Randomised Controlled Trial).** *PLoS One* 2015, **10:**e0140662.
187. Yu SY, Lee TJ, Jang SH, Han JW, Kim TH, Kim KW: **Cost-effectiveness of nationwide opportunistic screening program for dementia in South Korea.** *J Alzheimers Dis* 2015, **44:**195-204.
188. Heuzenroeder L, Donnelly M, Haby MM, Mihalopoulos C, Rossell R, Carter R, Andrews G, Vos T: **Cost-effectiveness of psychological and pharmacological interventions for generalized anxiety disorder and panic disorder.** *Australian and New Zealand Journal of Psychiatry* 2004, **38:**602-612.
189. Nosyk B, Guh DP, Bansback NJ, Oviedo-Joekes E, Brissette S, Marsh DC, Meikleham E, Schechter MT, Anis AH: **Cost-effectiveness of diacetylmorphine versus methadone for chronic opioid dependence refractory to treatment.** *CMAJ* 2012, **184:**E317-328.
190. Taylor M, Leonardi-Bee J, Agboola S, McNeill A, Coleman T: **Cost effectiveness of interventions to reduce relapse to smoking following smoking cessation.** *Addiction* 2011, **106:**1819-1826.
191. Achana F, Sutton AJ, Kendrick D, Hayes M, Jones DR, Hubbard SJ, Cooper NJ: **A decision analytic model to investigate the cost-effectiveness of poisoning prevention practices in households with young children.** *BMC Public Health* 2016, **15:**705.
192. Glotzer DE, Freedberg KA, Bauchner H: **Management of childhood lead poisoning: clinical impact and cost-effectiveness.** *Med Decis Making* 1995, **15:**13-24.
193. Murphy SM, Campbell AN, Ghitza UE, Kyle TL, Bailey GL, Nunes EV, Polsky D: **Cost-effectiveness of an internet-delivered treatment for substance abuse: Data from a multisite randomized controlled trial.** *Drug Alcohol Depend* 2016, **161:**119-126.
194. Alkire BC, Vincent JR, Burns CT, Metzler IS, Farmer PE, Meara JG: **Obstructed labor and caesarean delivery: the cost and benefit of surgical intervention.** *PLoS One* 2012, **7:**e34595.
195. Sculpher M, Manca A, Abbott J, Fountain J, Mason S, Garry R: **Cost effectiveness analysis of laparoscopic hysterectomy compared with standard hysterectomy: results from a randomised trial.** *BMJ* 2004, **328:**134.
196. Hullfish KL, Trowbridge ER, Stukenborg GJ: **Treatment strategies for pelvic organ prolapse: a cost-effectiveness analysis.** *Int Urogynecol J* 2011, **22:**507-515.
197. Epiu I, Alia G, Mukisa J, Tavrow P, Lamorde M, Kuznik A: **Estimating the cost and cost-effectiveness for obstetric fistula repair in hospitals in Uganda: a low income country.** *Health Policy Plan* 2018, **33:**999-1008.
198. Chanthavilay P, Reinharz D, Mayxay M, Phongsavan K, Marsden DE, Moore L, White LJ: **Economic Evaluation of Screening Strategies Combined with HPV Vaccination of Preadolescent Girls for the Prevention of Cervical Cancer in Vientiane, Lao PDR.** *PLoS One* 2016, **11:**e0162915.
199. Havrilesky LJ, Maxwell GL, Myers ER: **Cost-effectiveness analysis of annual screening strategies for endometrial cancer.** *Am J Obstet Gynecol* 2009, **200:**640 e641-648.
200. Zowall H, Cairns JA, Brewer C, Lamping DL, Gedroyc WM, Regan L: **Cost-effectiveness of magnetic resonance-guided focused ultrasound surgery for treatment of uterine fibroids.** *BJOG* 2008, **115:**653-662.

201. Yang KY, Caughey AB, Little SE, Cheung MK, Chen LM: **A cost-effectiveness analysis of prophylactic surgery versus gynecologic surveillance for women from hereditary non-polyposis colorectal cancer (HNPCC) Families.** *Fam Cancer* 2011, **10:**535-543.
202. Campos NG, Maza M, Alfaro K, Gage JC, Castle PE, Felix JC, Cremer ML, Kim JJ: **The comparative and cost-effectiveness of HPV-based cervical cancer screening algorithms in El Salvador.** *Int J Cancer* 2015, **137:**893-902.
203. Cowett AA, Golub RM, Grobman WA: **Cost-effectiveness of dilation and evacuation versus the induction of labor for second-trimester pregnancy termination.** *Am J Obstet Gynecol* 2006, **194:**768-773.
204. Roberts G, Roberts C, Jamieson A, Grimes C, Conn G, Bleichrodt R: **Surgery and Obstetric Care are Highly Cost-Effective Interventions in a Sub-Saharan African District Hospital: A Three-Month Single-Institution Study of Surgical Costs and Outcomes.** *World J Surg* 2016, **40:**14-20.
205. Clegg JP, Guest JF: **Modelling the cost-utility of bio-electric stimulation therapy compared to standard care in the treatment of elderly patients with chronic non-healing wounds in the UK.** *Curr Med Res Opin* 2007, **23:**871-883.
206. Cox CE, Carson SS, Govert JA, Chelluri L, Sanders GD: **An economic evaluation of prolonged mechanical ventilation.** *Crit Care Med* 2007, **35:**1918-1927.
207. Puri V, Pyrdeck TL, Crabtree TD, Kreisel D, Krupnick AS, Colditz GA, Patterson GA, Meyers BF: **Treatment of malignant pleural effusion: a cost-effectiveness analysis.** *Ann Thorac Surg* 2012, **94:**374-379; discussion 379-380.
208. Hall MK, Omer T, Moore CL, Taylor RA: **Cost-effectiveness of the Cardiac Component of the Focused Assessment of Sonography in Trauma Examination in Blunt Trauma.** *Acad Emerg Med* 2016, **23:**415-423.
209. Brasel KJ, Borgstrom DC, Weigelt JA: **Management of penetrating colon trauma: A cost-utility analysis.** *Surgery* 1999, **125:**471-479.
210. Slover J, Hoffman MV, Malchau H, Tosteson AN, Koval KJ: **A cost-effectiveness analysis of the arthroplasty options for displaced femoral neck fractures in the active, healthy, elderly population.** *J Arthroplasty* 2009, **24:**854-860.
211. Rosas SE, Feldman HI: **Synthetic vascular hemodialysis access versus native arteriovenous fistula: a cost-utility analysis.** *Ann Surg* 2012, **255:**181-186.
212. Nwachukwu BU, So C, Schairer WW, Shubin Stein BE, Strickland SM, Green DW, Dodwell ER: **Economic Decision Model for First-Time Traumatic Patellar Dislocations in Adolescents.** *Am J Sports Med* 2017, **45:**2267-2275.
213. Krishnan NM, Chatterjee A, Rosenkranz KM, Powell SG, Nigriny JF, Vidal DC: **The cost effectiveness of acellular dermal matrix in expander-implant immediate breast reconstruction.** *J Plast Reconstr Aesthet Surg* 2014, **67:**468-476.
214. Tadisina KK, Chopra K, Tangredi J, Thomson JG, Singh DP: **Helping hands: a cost-effectiveness study of a humanitarian hand surgery mission.** *Plast Surg Int* 2014, **2014:**921625.
215. Heard C, Chaboyer W, Anderson V, Gillespie BM, Whitty JA: **Cost-effectiveness analysis alongside a pilot study of prophylactic negative pressure wound therapy.** *J Tissue Viability* 2017, **26:**79-84.
216. Warf BC, Alkire BC, Bhai S, Hughes C, Schiff SJ, Vincent JR, Meara JG: **Costs and benefits of neurosurgical intervention for infant hydrocephalus in sub-Saharan Africa.** *J Neurosurg Pediatr* 2011, **8:**509-521.
217. Truzzi JC, Teich V, Pepe C: **Can hydrophilic coated catheters be beneficial for the public healthcare system in Brazil? - A cost-effectiveness analysis in patients with spinal cord injuries.** *Int Braz J Urol* 2018, **44:**121-131.
218. Green DA, Rink M, Cha EK, Xylinas E, Chughtai B, Scherr DS, Shariat SF, Lee RK: **Cost-effective treatment of low-risk carcinoma not invading bladder muscle.** *BJU Int* 2013, **111:**E78-84.
219. Bucher BT, Hall BL, Warner BW, Keller MS: **Intussusception in children: cost-effectiveness of ultrasound vs diagnostic contrast enema.** *J Pediatr Surg* 2011, **46:**1099-1105.
220. Shillcutt SD, Sanders DL, Teresa Butron-Vila M, Kingsnorth AN: **Cost-effectiveness of inguinal hernia surgery in northwestern Ecuador.** *World J Surg* 2013, **37:**32-41.
221. Govindarajan A, Naimark D, Coburn NG, Smith AJ, Law CH: **Use of colonic stents in emergent malignant left colonic obstruction: a Markov chain Monte Carlo decision analysis.** *Dis Colon Rectum* 2007, **50:**1811-1824.

222. Hamze H, Mengiste A, Carter J: **The impact and cost-effectiveness of the Amref Health Africa-Smile Train Cleft Lip and Palate Surgical Repair Programme in Eastern and Central Africa.** *Pan Afr Med J* 2017, **28:**35.
223. Kameda M, Yamada S, Atsuchi M, Kimura T, Kazui H, Miyajima M, Mori E, Ishikawa M, Date I, Sinphoni, Investigators S-: **Cost-effectiveness analysis of shunt surgery for idiopathic normal pressure hydrocephalus based on the SINPHONI and SINPHONI-2 trials.** *Acta Neurochir (Wien)* 2017, **159:**995-1003.
224. Zendejas B, Moriarty JP, O'Byrne J, Degnim AC, Farley DR, Boughey JC: **Cost-effectiveness of contralateral prophylactic mastectomy versus routine surveillance in patients with unilateral breast cancer.** *J Clin Oncol* 2011, **29:**2993-3000.
225. Zanocco K, Heller M, Elaraj D, Sturgeon C: **Is subtotal thyroidectomy a cost-effective treatment for Graves disease? A cost-effectiveness analysis of the medical and surgical treatment options.** *Surgery* 2012, **152:**164-172.
226. Panca M, Viner RM, White B, Pandya T, Melo H, Adamo M, Batterham R, Christie D, Kinra S, Morris S: **Cost-effectiveness of bariatric surgery in adolescents with severe obesity in the UK.** *Clin Obes* 2018, **8:**105-113.
227. Kent MS, Korn P, Port JL, Lee PC, Altorki NK, Korst RJ: **Cost effectiveness of chest computed tomography after lung cancer resection: a decision analysis model.** *Ann Thorac Surg* 2005, **80:**1215-1222; discussion 1222-1213.
228. Slover J, Espehaug B, Havelin LI, Engesaeter LB, Furnes O, Tomek I, Tosteson A: **Cost-effectiveness of unicompartmental and total knee arthroplasty in elderly low-demand patients. A Markov decision analysis.** *J Bone Joint Surg Am* 2006, **88:**2348-2355.
229. Chen AT, Pedtke A, Kobs JK, Edwards GS, Jr., Coughlin RR, Gosselin RA: **Volunteer orthopedic surgical trips in Nicaragua: a cost-effectiveness evaluation.** *World J Surg* 2012, **36:**2802-2808.
230. Taleban S, Van Oijen MG, Vasiliauskas EA, Fleshner PR, Shen B, Ippoliti AF, Targan SR, Melmed GY: **Colectomy with Permanent End Ileostomy Is More Cost-Effective than Ileal Pouch-Anal Anastomosis for Crohn's Colitis.** *Dig Dis Sci* 2016, **61:**550-559.
231. Park KT, Tsai R, Perez F, Cipriano LE, Bass D, Garber AM: **Cost-effectiveness of early colectomy with ileal pouch-anal anastamosis versus standard medical therapy in severe ulcerative colitis.** *Ann Surg* 2012, **256:**117-124.
232. Tan EK, Jacovides M, Khullar V, Teoh TG, Fernando RJ, Tekkis PP: **A cost-effectiveness analysis of delayed sphincteroplasty for anal sphincter injury.** *Colorectal Dis* 2008, **10:**653-662.
233. Hong NJ, Clarke GM, Yaffe MJ, Holloway CM: **Cost-effectiveness analysis of whole-mount pathology processing for patients with early breast cancer undergoing breast conservation.** *Curr Oncol* 2016, **23:**S23-31.
234. Kok NF, Adang EM, Hansson BM, Dooper IM, Weimar W, van der Wilt GJ, Ijzermans JN: **Cost effectiveness of laparoscopic versus mini-incision open donor nephrectomy: a randomized study.** *Transplantation* 2007, **83:**1582-1587.
235. Burfeind WR, Jr., Jaik NP, Villamizar N, Toloza EM, Harpole DH, Jr., D'Amico TA: **A cost-minimisation analysis of lobectomy: thoracoscopic versus posterolateral thoracotomy.** *Eur J Cardiothorac Surg* 2010, **37:**827-832.
236. Ferguson MK, Lehman AG: **Sleeve lobectomy or pneumonectomy: optimal management strategy using decision analysis techniques.** *Ann Thorac Surg* 2003, **76:**1782-1788.
237. Wong CK, Lang BH: **A cost-utility analysis for prophylactic central neck dissection in clinically nodal-negative papillary thyroid carcinoma.** *Ann Surg Oncol* 2014, **21:**767-777.
238. Thomas JA, Tubaro A, Barber N, Thorpe A, Armstrong N, Bachmann A, Van Hout B: **The Continuing Story of the Cost-Effectiveness of Photoselective Vaporization of the Prostate versus Transuretheral Resection of the Prostate for the Treatment of Symptomatic Benign Prostatic Obstruction.** *Value Health* 2015, **18:**376-386.
239. Yiee JH, Baskin LS: **Use of internal stent, external transanastomotic stent or no stent during pediatric pyeloplasty: a decision tree cost-effectiveness analysis.** *J Urol* 2011, **185:**673-680.
240. Kulkarni GS, Alibhai SM, Finelli A, Fleshner NE, Jewett MA, Lopushinsky SR, Bayoumi AM: **Cost-effectiveness analysis of immediate radical cystectomy versus intravesical Bacillus Calmette-Guerin therapy for high-risk, high-grade (T1G3) bladder cancer.** *Cancer* 2009, **115:**5450-5459.

241. Torquati A, Lutfi R, Khaitan L, Sharp KW, Richards WO: **Heller myotomy vs Heller myotomy plus Dor fundoplication: cost-utility analysis of a randomized trial.** *Surg Endosc* 2006, **20:**389-393.
242. Deppen SA, Davis WT, Green EA, Rickman O, Aldrich MC, Fletcher S, Putnam JB, Jr., Grogan EL: **Cost-effectiveness of initial diagnostic strategies for pulmonary nodules presenting to thoracic surgeons.** *Ann Thorac Surg* 2014, **98:**1214-1222.
243. Morton RL, Howard K, Thompson JF: **The cost-effectiveness of sentinel node biopsy in patients with intermediate thickness primary cutaneous melanoma.** *Ann Surg Oncol* 2009, **16:**929-940.
244. Javanbakht M, Azuara-Blanco A, Burr JM, Ramsay C, Cooper D, Cochran C, Norrie J, Scotland G: **Early lens extraction with intraocular lens implantation for the treatment of primary angle closure glaucoma: an economic evaluation based on data from the EAGLE trial.** *BMJ Open* 2017, **7:**e013254.
245. Leung VC, Pechlivanoglou P, Chew HF, Hatch W: **Corneal Collagen Cross-Linking in the Management of Keratoconus in Canada: A Cost-Effectiveness Analysis.** *Ophthalmology* 2017, **124:**1108-1119.
246. Hirneiss C, Neubauer AS, Niedermeier A, Messmer EM, Ulbig M, Kampik A: **Cost utility for penetrating keratoplasty in patients with poor binocular vision.** *Ophthalmology* 2006, **113:**2176-2180.
247. Beauchamp CL, Beauchamp GR, Stager DR, Sr., Brown MM, Brown GC, Felius J: **The cost utility of strabismus surgery in adults.** *J AAPOS* 2006, **10:**394-399.
248. Chang JS, Smiddy WE: **Cost-effectiveness of retinal detachment repair.** *Ophthalmology* 2014, **121:**946-951.
249. Moriarty JP, Borah BJ, Foote RL, Pulido JS, Shah ND: **Cost-effectiveness of proton beam therapy for intraocular melanoma.** *PLoS One* 2015, **10:**e0127814.
250. Busbee BG, Brown MM, Brown GC, Sharma S: **CME review: A cost-utility analysis of laser photocoagulation for extrafoveal choroidal neovascularization.** *Retina* 2003, **23:**279-287; ; quiz 443-274.
251. Rudmik L, Smith KA, Kilty S: **Endoscopic polypectomy in the clinic: a pilot cost-effectiveness analysis.** *Clin Otolaryngol* 2016, **41:**110-117.
252. Rudmik L, Soler ZM, Mace JC, Schlosser RJ, Smith TL: **Economic evaluation of endoscopic sinus surgery versus continued medical therapy for refractory chronic rhinosinusitis.** *Laryngoscope* 2015, **125:**25-32.
253. Tseng C-C, Lai M-T, Wu C-C, Yuan S-P, Ding Y-F: **Cost-effectiveness analysis of endoscopic tympanoplasty versus microscopic tympanoplasty for chronic otitis media in Taiwan.** *JCMA Journal of the Chinese Medical Association* 2018, **81:**284-290.
254. Ward MJ, Bonomo JB, Adeoye O, Raja AS, Pines JM: **Cost-effectiveness of diagnostic strategies for evaluation of suspected subarachnoid hemorrhage in the emergency department.** *Acad Emerg Med* 2012, **19:**1134-1144.
255. Manchikanti L, Falco FJ, Pampati V, Cash KA, Benyamin RM, Hirsch JA: **Cost utility analysis of caudal epidural injections in the treatment of lumbar disc herniation, axial or discogenic low back pain, central spinal stenosis, and post lumbar surgery syndrome.** *Pain Physician* 2013, **16:**E129-143.
256. Mwamburi M, Tenaglia AT, Leibler EJ, Staats PS: **Cost-effectiveness of noninvasive vagus nerve stimulation for acute treatment of episodic migraine and role in treatment sequence strategies.** *Am J Manag Care* 2018, **24:**S527-S533.
257. Mohr NM, Stoltze A, Ahmed A, Kiscaden E, Shane D: **Using continuous quantitative capnography for emergency department procedural sedation: a systematic review and cost-effectiveness analysis.** *Intern Emerg Med* 2018, **13:**75-85.
258. Feller-Kopman D, Liu S, Geisler BP, DeCamp MM, Pietzsch JB: **Cost-Effectiveness of a Bronchial Genomic Classifier for the Diagnostic Evaluation of Lung Cancer.** *J Thorac Oncol* 2017, **12:**1223-1232.
259. Borghi J, Guinness L, Ouedraogo J, Curtis V: **Is hygiene promotion cost-effective? A case study in Burkina Faso.** *Trop Med Int Health* 2002, **7:**960-969.
260. Shrestha RK, Marseille E, Kahn JG, Lule JR, Pitter C, Blandford JM, Bunnell R, Coutinho A, Kizito F, Quick R, Mermin J: **Cost-effectiveness of home-based chlorination and safe water storage in reducing diarrhea among HIV-affected households in rural Uganda.** *Am J Trop Med Hyg* 2006, **74:**884-890.
261. Manasyan A, Chomba E, McClure EM, Wright LL, Krzywanski S, Carlo WA, Eunice Kennedy Shriver National Institute of Child H, Human Development Global Network for Ws, Children's Health R: **Cost-effectiveness of essential newborn care training in urban first-level facilities.** *Pediatrics* 2011, **127:**e1176-1181.

262. Dalziel K, Segal L, Katz R: **Cost-effectiveness of mandatory folate fortification v. other options for the prevention of neural tube defects: results from Australia and New Zealand.** *Public Health Nutr* 2010, **13:**566-578.
263. Rahman F, Bose S, Linnan M, Rahman A, Mashreky S, Haaland B, Finkelstein E: **Cost-effectiveness of an injury and drowning prevention program in Bangladesh.** *Pediatrics* 2012, **130:**e1621-1628.
264. Bertram MY, Lim SS, Barendregt JJ, Vos T: **Assessing the cost-effectiveness of drug and lifestyle intervention following opportunistic screening for pre-diabetes in primary care.** *Diabetologia* 2010, **53:**875-881.
265. Townsend J, Greenland K, Curtis V: **Costs of diarrhoea and acute respiratory infection attributable to not handwashing: the cases of India and China.** *Trop Med Int Health* 2017, **22:**74-81.
266. Rubinstein A, Garcia Marti S, Souto A, Ferrante D, Augustovski F: **Generalized cost-effectiveness analysis of a package of interventions to reduce cardiovascular disease in Buenos Aires, Argentina.** *Cost Eff Resour Alloc* 2009, **7:**10.
267. Ngalesoni F, Ruhago G, Mayige M, Oliveira TC, Robberstad B, Norheim OF, Higashi H: **Cost-effectiveness analysis of population-based tobacco control strategies in the prevention of cardiovascular diseases in Tanzania.** *PLOS ONE* 2017, **12:**e0182113.
268. Dalziel K, Segal L: **Time to give nutrition interventions a higher profile: cost-effectiveness of 10 nutrition interventions.** *Health Promot Int* 2007, **22:**271-283.
